# An Interpolation Method to Reduce the Computational Time in the Stochastic Lagrangian Particle Dispersion Modeling of Spatially Dense XCO_2_ Retrievals

**DOI:** 10.1029/2020EA001343

**Published:** 2021-04-02

**Authors:** Dustin Roten, Dien Wu, Benjamin Fasoli, Tomohiro Oda, John C. Lin

**Affiliations:** ^1^ Department of Atmospheric Sciences University of Utah Salt Lake City UT USA; ^2^ Division of Geological and Planetary Sciences California Institute of Technology Pasadena CA USA; ^3^ Goddard Earth Sciences Technology and Research Universities Space Research Association Columbia MD USA; ^4^ NASA Goddard Space Flight Center Global Modeling and Assimilation Office Greenbelt MD USA

**Keywords:** interpolation, Lagrangian particle dispersion modeling, land‐atmosphere Interactions, orbiting carbon observatory, space‐based CO_2_ observations, X‐STILT

## Abstract

A growing constellation of satellites is providing near‐global coverage of column‐averaged CO_2_ observations. Launched in 2019, NASA’s OCO‐3 instrument is set to provide XCO_2_ observations at a high spatial and temporal resolution for regional domains (100 × 100 km). The atmospheric column version of the Stochastic Time‐Inverted Lagrangian Transport (X‐STILT) model is an established method of determining the influence of upwind sources on column measurements of the atmosphere, providing a means of analysis for current OCO‐3 observations and future space‐based column‐observing missions. However, OCO‐3 is expected to provide hundreds of soundings per targeted observation, straining this already computationally intensive technique. This work proposes a novel scheme to be used with the X‐STILT model to generate upwind influence footprints with less computational expense. The method uses X‐STILT generated influence footprints from a key subset of OCO‐3 soundings. A nonlinear weighted averaging is applied to these footprints to construct additional footprints for the remaining soundings. The effects of subset selection, meteorological data, and topography are investigated for two test sites: Los Angeles, California, and Salt Lake City, Utah. The computational time required to model the source sensitivities for OCO‐3 interpretation was reduced by 62% and 78% with errors smaller than other previously acknowledged uncertainties in the modeling system (OCO‐3 retrieval error, atmospheric transport error, prior emissions error, etc.). Limitations and future applications for future CO_2_ missions are also discussed.

## Key Points

- Determining sources of spatially dense XCO_2_ observations with LPDM techniques can become time intensive and strain computational resources
- Presented in this work is an interpolation scheme that eases the computational burden of spatially dense XCO_2_ source determination studies
- Evaluating the efficiency of this interpolation scheme revealed reductions of >50% in computational time at two testing locations

## 1 Introduction

As global concentrations of carbon dioxide pass the 400 ppm milestone (Keeling & Keeling, 2017), the focus on its mitigation has increased at the local, national, and international levels, guiding the implementation of several policies. Examples of such policies include the Kyoto Protocol (2005) and Paris Climate Agreement (2016), developed and implemented at the international level. Of myriad CO_2_ sinks and sources that exist, emissions from urban areas are of particular interest as they are responsible for 40%–70% of the total CO_2_ emitted (United Nations Human Settlements Programme, 2011). Thus, several local‐level policies have been implemented to address the CO_2_ emissions from cities, one notable example being the C40 city network (Davidson et al., 2019). Since monitoring the CO_2_ fluxes of these urban areas is of scientific and political importance, research efforts have equipped several cities with mobile and stationary ground‐based measurement networks.

Observational networks such as the Utah Urban Carbon Dioxide Network (UUCON) and mobile rail‐based (TRAX) measurements in Salt Lake City, UT (Bares et al., 2019; Mitchell, Crosman, et al., 2018), the Indianapolis Flux Experiment (INFLUX) in Indianapolis, IN (Richardson et al., 2017), the Berkeley Environmental Air Quality & CO_2_ Network (BEACON) in San Francisco, CA (Shusterman et al., 2016), the Los Angeles Megacity Carbon Project in Los Angeles, CA (Verhulst et al., 2017), and the Munich Urban Carbon Column Network (MUCCnet) in Munich, Germany (Dietrich et al., 2021) all provide insights into the spatiotemporal dynamics of these urban emissions. It is often the case that urban emissions produce atmospheric CO_2_ concentrations that are measurably higher than regional baseline values (Jacobson, 2010; Wang & Ostoja‐Starzewski, 2004). Several studies have linked these emission characteristics to urban areas’ residential and economic activity (Kunik et al., 2019; Lin et al., 2018; Mitchell, Lin, et al., 2018; Tian et al., 2019; Xueref‐Remy et al., 2018; Wu et al., 2020). Understanding these linkages may help steer local‐level policy development aimed at the mitigation of urban CO_2_ emissions.

Although ground‐based CO_2_ monitoring networks provide records at high temporal resolution, the spatial density of measurement sites is often sparse. The operation of these networks is costly, limiting their size and the number of cities instrumented with such platforms. Space‐based instrumentation can assess CO_2_ enhancements and extend knowledge from existing measurement networks to new cities around the world. At the sacrifice of measurement frequency, this type of instrumentation provides near‐global coverage of urban areas that may be lacking in ground‐based measurements (Yue et al., 2016). Currently, data from several space‐based CO_2_ observation platforms are available. Examples include the National Aeronautics and Space Administration’s (NASA) OCO‐2 instrument (Eldering et al., 2012; Wunch et al., 2017), the Chinese Academy of Sciences’ TanSat (Liu et al., 2018), and the Japan Aerospace Exploration Agency’s (JAXA) GOSAT‐I and GOSAT‐II instruments (Yokota et al., 2009).

Understanding the relationship between CO_2_ fluxes and atmospheric warming is a crucial step toward mitigating global climate change (Le Quéré et al., 2009; Matthews et al., 2014). Efforts to bolster CO_2_ monitoring have led to innovative methods for measuring atmospheric mixing ratios. Ground‐based stationary networks are becoming more dense and mobile measurement platforms are increasingly utilized to provide high‐resolution spatiotemporal CO_2_ measurements (Apte et al., 2017; Bush et al., 2015; Mitchell, Crosman, et al., 2018; Shusterman et al., 2016). Likewise, the number of space‐based instruments is set to increase in the near future. NASA’s Orbiting Carbon Observatory mission has recently added an additional instrument to its arsenal of space‐based observation platforms. Originally built as a spare to the OCO‐2 instrument, OCO‐3 was launched in May of 2019 and was successfully installed on the International Space Station (ISS). With its unique orbital path and “pointing mirror assembly” (PMA), this instrument is capable of performing dense scans of column‐averaged CO_2_ (XCO_2_) over specific urban areas (so called “snapshot area mapping,” or SAM). These SAMs consist of discretized atmospheric soundings in a rectangular region (∼100 km × ∼100 km) over key areas (Eldering et al., 2019). Compared to the 16‐days revisit time of OCO‐2, the revisit time of OCO‐3 is erratic. Its unique flight path and PMA provide revisits ranging from a few per year to multiple per day.

As with ground‐based measurements, space‐based observations may be coupled with gridded emission inventories to provide a means of validation through model and observation agreement (Hedelius et al., 2018; Janardanan et al., 2016; Labzovskii et al., 2019; Wu et al., 2018; Ye et al., 2020). This coupling often leverages Eulerian and Lagrangian atmospheric modeling techniques, which link emissions’ sources with observations of the atmosphere. In previous analyses, Wu et al. (2018) (referred to hereafter as W18) built upon the Stochastic Time‐Inverted Lagrangian Transport (STILT) model by extending the traditional surface‐based receptor scheme to a column‐based receptor scheme (Fasoli et al., 2018; Lin et al., 2003; Wu et al., 2018). This new configuration was applied to aggregated XCO_2_ values provided by OCO‐2 observations, producing a sensitivity matrix defining the upstream area contributing to mole fractions observed at the receptor location. This matrix, known as a “footprint,” was generated for multiple sounding locations and the contribution of nearby urban areas to detected XCO_2_ enhancements was quantified.

The W18 method couples satellite observations and emission inventories using modeling processes. The increase in observation density and more frequent revisit times provided by OCO‐3 is expected to make this method of analysis inefficient, as the amount of computational time will be greater than in previous work. To accommodate the increase in simulations required to interpret OCO‐3 measurements, this work presents an interpolation method capable of reducing resource requirements. This new technique is designed to work in tandem with the X‐STILT model or other inverse modeling framework where influence footprints are generated. In situations where a large number of spatially distributed, column‐based receptors are to be processed, this method systematically subsets the receptor field, generates a fraction of the influence footprints, then interpolates the remaining influence footprints. The interpolation method provides a faster means to footprint generation relative to the traditional X‐STILT method.

Presented here is a description of the interpolation process and an evaluation of its effectiveness. At the time of writing, preliminary OCO‐3 data were not fully quality assured; therefore, the presented methodology used a series of simulated SAMs (s‐SAMs) over two cities in the United States: Los Angeles, CA and Salt Lake City, UT. At both locations, each s‐SAM was compared to a set of interpolated counterparts in which the dependency on the interpolation scheme was systematically increased. Each iteration was compared to the original s‐SAM by quantifying the spatial distribution and point‐wise agreements between individual s‐SAM values and their respective footprints. Additional consideration was given to the influence of large CO_2_ sources (power generating and manufacturing plants, etc.) on this method’s ability to accurately produce XCO_2_ values using limited information. For improvements, a large point source detection algorithm was applied to s‐SAMs to reduce cases of large error. Since the X‐STILT model can be driven by a variety of meteorological data, the effects of this data’s resolution on the interpolation were also addressed. The overall utility of this method is then discussed in the context of monitoring urban CO_2_ emissions, the OCO‐3 instrument, and future space‐based missions.

## 2 Methods

### 2.1 Coupling X‐STILT and ODIAC

The X‐STILT model developed by W18 requires several user‐supplied parameters, three of which include: (1) the geolocation of each column‐based receptor, (2) meteorological fields to drive the model, and (3) the amount of time to propagate a backwards trajectory. SAMs provided by OCO‐3 are a set of near‐uniformly distributed soundings, each with a unique longitude and latitude (*λ*, *ϕ*). When applying the X‐STILT model, a column‐averaged influence footprint is generated for each sounding location. Each footprint, denoted as *F*(*λ*, *ϕ*, *t*_0_), is constructed by distributing a series of individual X‐STILT receptors at interval altitudes above the location (*λ*, *ϕ*) as prescribed by W18. Collectively, these vertical distributions constitute a column receptor. Incorporating turbulence, ensembles of particles released at each receptor location act as air parcels and follow the supplied meteorological fields backwards in time (beginning at time *t*_0_) across a grid (*x*, *y*) independent of the s‐SAM. W18’s full calculation required for the influence footprint is summarized in Equation 1:

(1) $$F\left(\lambda ,\phi ,t_{0}\;|\;x_{i},y_{j},t_{\xi }\right)=\left(\frac{m_{air}}{h\bar{\rho }\left(x_{i},y_{j},t_{\xi }\right)}\frac{1}{N}\overset{N}{\underset{p=1}{\sum }}\Delta t_{p,i,j,z\leq h}\right)\times A_{n}\times P_{n}.$$

The contribution of each particle/parcel released from the column‐based receptor is summed. This contribution is defined as the amount of time a particle’s (*p*) trajectory propagates below a mixing layer (*z* ≤ *h*) for each backward time step, *ξ*, over each grid space (*x*_*i*_, *y*_*j*_). This calculation is performed for all particles, *N*. Other parameters are the dry‐air mass, *m*_*air*_, and the mean atmospheric density (for *z* ≤ *h*), $\bar{\rho }$, at grid cell (*x*_*i*_, *y*_*j*_). Equation 1 represents a column‐based footprint and therefore requires a vertical weighting throughout the atmospheric column. A weighting scheme is given to the X‐STILT model by providing the averaging kernel and pressure weighting function *A*_*n*_ and *P*_*n*_ from OCO‐2 sounding data. Vertical release locations within the column receptor are indicated by *n*. After temporal integration and vertical averaging, each footprint is represented by a matrix of values reflecting the amount of influence (in ppm) per surface flux (μmol/m^2^/s) a particular location and start time has on the column‐averaged value. For simplicity of notation in the discussions that follow, *F*(*λ*, *ϕ*, *t*_0_) will represent the footprint associated with a particular column receptor at (*λ*, *ϕ*) and *f* (*x*_*i*_, *y*_*j*_, *t*_0_) will represent its distributed elements of influence values.

**Figure 1:**
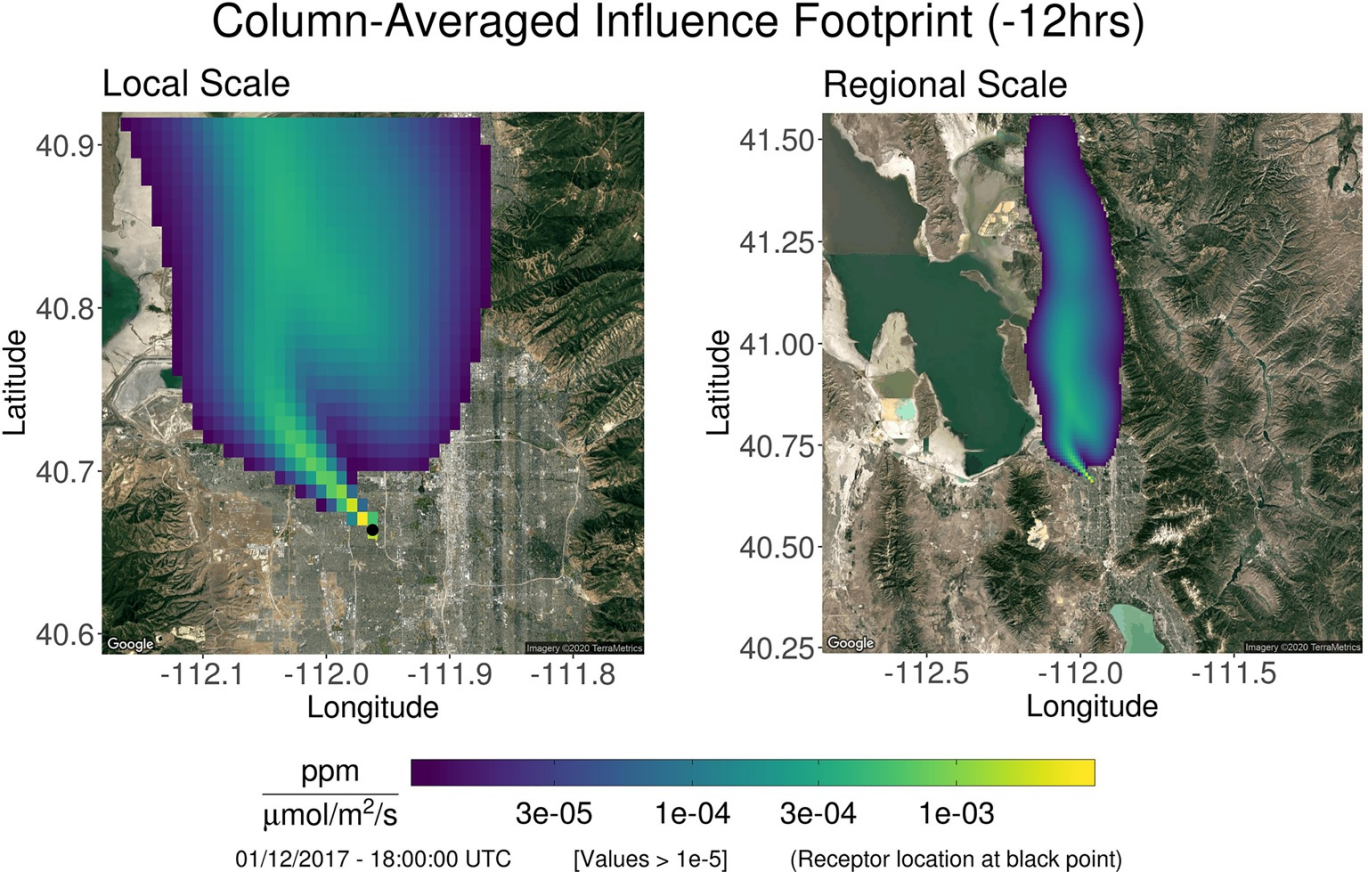
The X‐STILT model was used to produce a footprint for a column‐based receptor placed within the Salt Lake Valley (black point; left panel) and was initialized for time 18:00:00 UTC on January 12, 2017. Using 3 km HRRR data, particles were driven backwards in time for 12 hours and temporally integrated. Examining the footprint on a regional scale (right panel) reveals strong northerly winds. It should be noted that only footprint values ≥ 10^−5^ ppm/(μmol/m^2^/s) were included. Nonfiltered footprints can be more expansive than what is presented here. (X‐STILT, Stochastic Time‐Inverted Lagrangian Transport).

As an example, Figure 1 presents a column‐based footprint generated by the X‐STILT model within the Salt Lake Valley. Backwards‐in‐time trajectories were calculated for 12 hours beginning at 18:00:00 UTC on January 12, 2017. The column receptor from which the particle trajectories originate is located at the black point (left panel) and is identified as *F*(*λ*, *ϕ*, *t*_0_). The cells making up the footprint lie on the (*x*, *y*) grid and represent each location’s influence (per surface flux) on the “observation” made at the receptor. These values are denoted as *f* (*x*_*i*_, *y*_*j*_, *t*_0_). As released particles propagate backwards in time, the sensitivity to surface fluxes typically decays as the distance from the column receptor is increased. The footprint’s depiction on a regional scale (right panel) demonstrates the northerly winds experienced by the region during this time. The s‐SAMs generated in this work consist of many column receptors equally spaced on a grid to emulate OCO‐3’s measurement capabilities. Column receptors and their footprints are independently generated and do not influence one another.

A priori column‐based receptor values can be calculated by convolving footprints with surface emission inventories. This work used the 2019 release of the Open‐source Data Inventory for Anthropogenic CO_2_ (ODIAC), a spatially explicit 1 × 1 km inventory that uses the locations of power plants and nighttime light density to approximate anthropogenic CO_2_ fluxes (Oda et al., 2018; Oda & Maksyutov, 2011). This inventory is resolved at the month level and therefore is represented as a temporally static field, Φ(*x*_*i*_, *y*_*j*_), as the length of typical backwards trajectories span from a few hours to a few days. Calculating an XCO_2_ enhancement due to this anthropogenic release can be performed by an element‐wise multiplication (Hadamard product) of these two fields followed by an element‐wise summation (Equation 2):

(2) $$\Delta {XCO}_{2}\left(\lambda ,\phi ,t_{0}\right)=\underset{i,j}{\sum }F\left(\lambda ,\phi ,t_{0}\;|\;x_{i},y_{j},t_{\xi }\right)○\Phi \left(x_{i},y_{j}\right)$$

**Figure 2:**
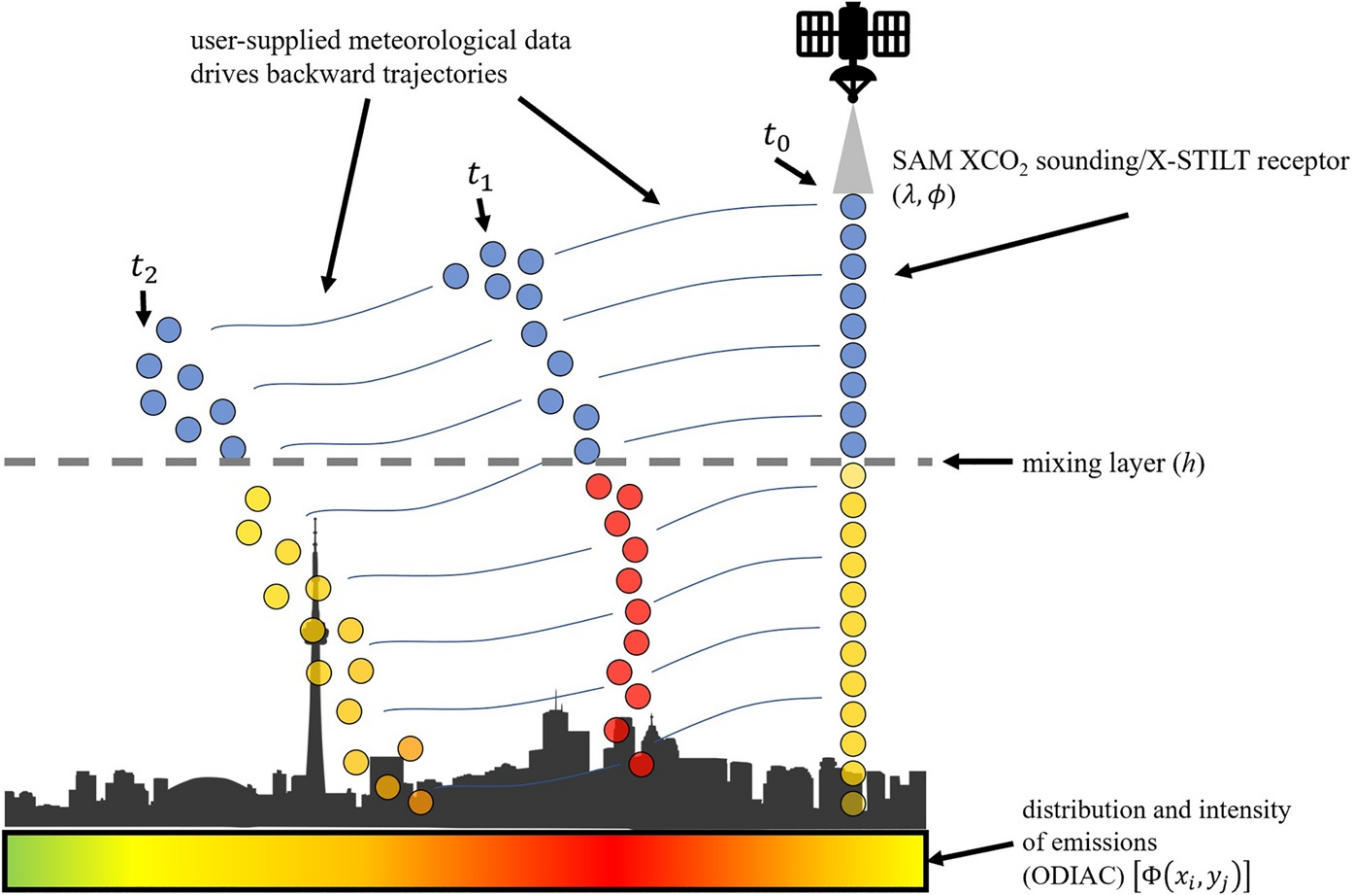
An atmospheric sounding obtained during an OCO‐3 transect provides a column‐averaged CO_2_ value. Its location is passed to the X‐STILT model and a series of receptors are vertically distributed throughout the atmospheric column. X‐STILT then uses the supplied meteorological data to propagate particles backwards in time through multiple time steps (*t*_1_, *t*_2_, …) across an independent domain gridded by user specifications. Particles’ locations and times spent below the mixing layer (*h*) indicates the magnitude of influence each grid cell had on the overall column‐averaged value recorded at the receptor [units: ppm/(μmol/m^2^/s)]. These point‐wise influences can be multiplied by the corresponding grid cell in ODIAC [units: μmol/m^2^/s] to determine the contribution (in ppm) of a specific location to the total column concentration. ODIAC reflects surface CO_2_ fluxes related to large point sources and urban density. Areas of low flux are represented in green while higher flux is represented by red. This color scheme represents the urban density associated with the black silhouette. Particles with heights below the mixing layer (*z*_*p*_ < *h*) interact with ODIAC grid cells. Here, they are assigned the color of associated surface flux value. X‐STILT applications release many more particles from receptor locations than what is depicted here. (OCO, Orbiting Carbon Observatory; X‐STILT, Stochastic Time‐Inverted Lagrangian Transport; ODIAC, Open‐source Data Inventory for Anthropogenic CO_2_).

The calculated XCO_2_ enhancement, ΔXCO_2_, represents the amount added to the regional background XCO_2_ value and typically falls between 0ppm and 10ppm. A simplified application of X‐STILT is presented in Figure 2 in which a single column‐averaged sounding is analyzed. Also depicted is a representation of how ODIAC fluxes reflect urban density and human‐related emissions. For further details on the evaluation and functionality of X‐STILT, the reader is referred to the following works: Lin (2003), Fasoli et al. (2018), and Wu et al. (2018).

For this work, X‐STILT parameter values were selected from W18 such that each column‐based receptor initially consisted of individual receptors distributed vertically upward and spaced 100 m apart. Receptors with this spacing characteristic covered 3 km above ground level (AGL). Beyond 3 km, receptors maintained a 500 m spacing. The maximum height for all column‐based receptors was 6 km AGL. Each receptor in the column released 100 particles per 2 min time step, driven by the 3 km High‐Resolution Rapid Refresh (HRRR) model (Benjamin et al., 2016; Rolph et al., 2017) for a total of 12 hours backwards in time. The result was integrated in time and the footprints from each vertically placed receptor were spatially averaged to construct the column‐based footprint. W18 investigated the output’s sensitivity to the selection of these parameters and reported ∼4% error when compared to observations. Values for *A*_*n*_ and *P*_*n*_ were gathered from the closest available OCO‐2 sounding in both space and time (OCO‐2 data can be downloaded at https://disc.gsfc.nasa.gov/).

### 2.2 Snapshot Area Mapping and Target Locations

**Figure 3:**
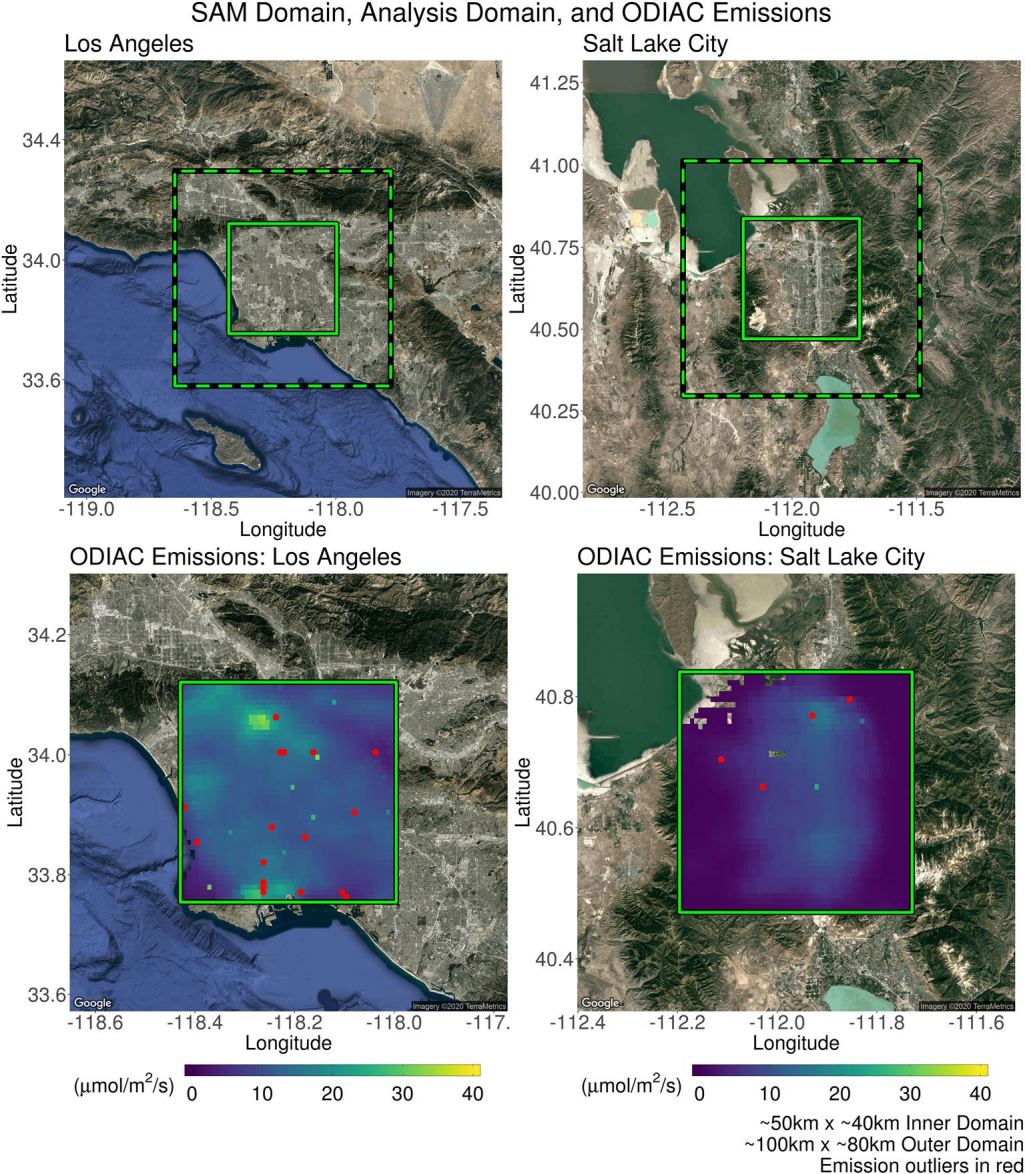
(Top row) The domain indicated by the green dashed box is representative of the ∼100 × ∼100 km domain that characterizes the spatial extent of SAMs supplied by the OCO‐3 instrument. The smaller inner domain depicts the spatial coverage of the generated s‐SAMs which includes the greater metropolitan area of each test location. Dimensions of the inner domains are 48 × 41 km and 52 × 41 km for Los Angeles and Salt Lake City, respectively. OCO‐3 supplied SAMs may have domains rotated about their center and are not necessarily aligned as presented here. (Bottom row) ODIAC fluxes from January to June of 2017 are averaged and presented within the context of s‐SAM domains. Large CO_2_ flux sources (>50 μmol/m^2^/s), indicated by red points, correspond to power plants or other large industrial complexes. (SAM, snapshot area mapping; OCO, Orbiting Carbon Observatory; ODIAC, Open‐source Data Inventory for Anthropogenic CO_2_).

Two locations within the western United States were selected as testing sites for the evaluation of the interpolation scheme: Los Angeles (LA), California and Salt Lake City (SLC), Utah. Both sites have distinctive topological and meteorological features that affect its performance. Los Angeles is a coastal city, bordered by the Pacific Ocean along the southwest. To the north and northwest sit Angeles National Forest and Mt. San Antonio. The borders of Angeles National Forest contain mountainous terrain and the prominence of Mt. San Antonio is roughly 1.9 km. The U.S. Census Bureau’s *Annual Estimates of the Residential Population (April 1, 2010 to July 1, 2018)* estimates Los Angeles’ greater metropolitan population to be just above 13.2 million (2017). Similarly, Salt Lake City sits in a valley among several mountain ranges. Immediately to the west are the Oquirrh Mountains, with a prominence of 1.6 km. The Wasatch mountains sit to the east with a prominence of 2.2 km. To the south are the smaller Traverse Mountains (prominence: 0.3 km) and to the northwest is the Great Salt Lake. U.S. Census estimates of the greater metropolitan population of Salt Lake City is 1.2 million (2017). Satellite images of both target locations are included in Figure 3 (Kahle & Wickham, 2013).

**Figure 4:**
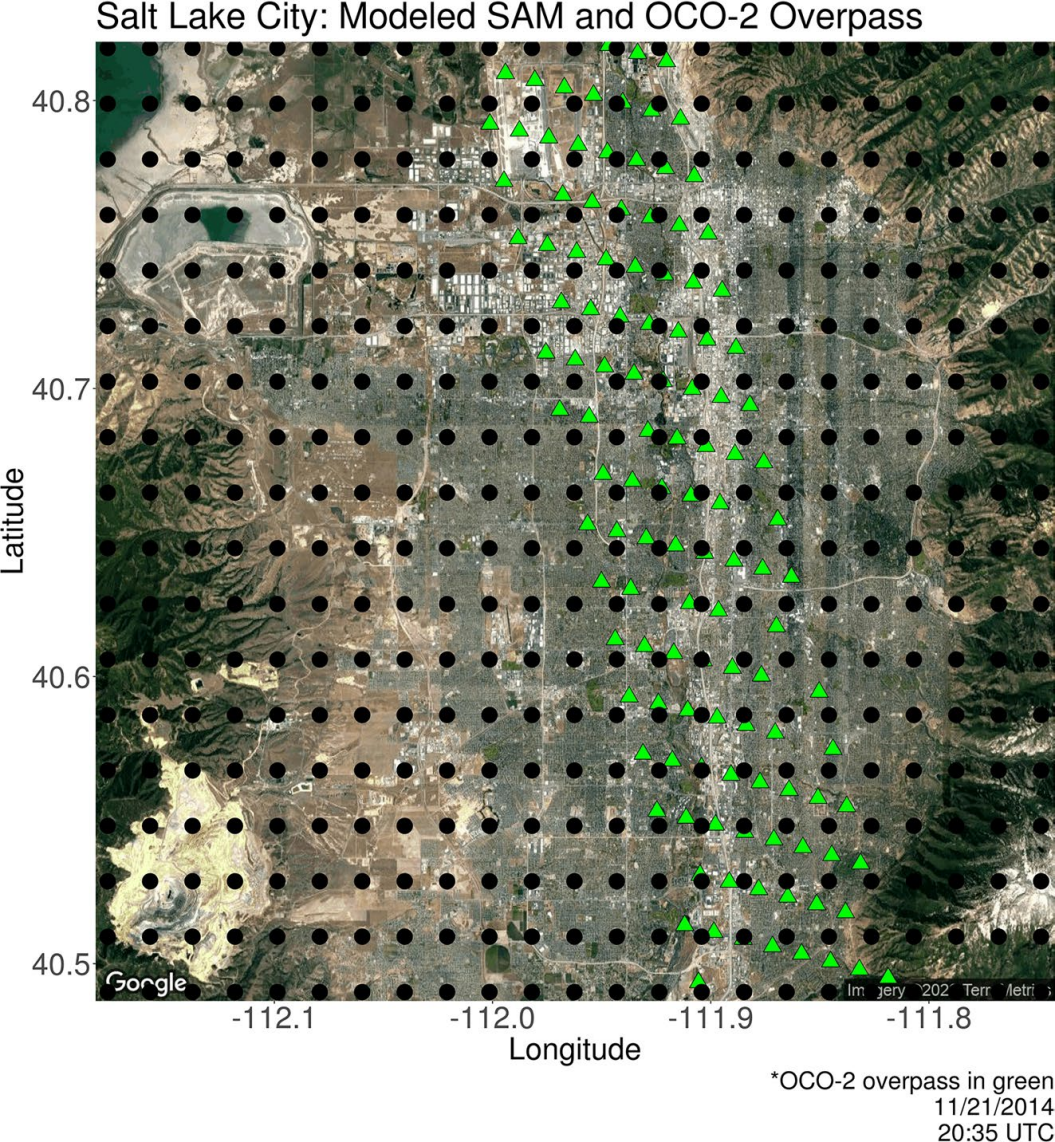
The distribution of soundings (0.019° × 0.019° ) that make up the s‐SAMs over Salt Lake City (black points) are accompanied with an example OCO‐2 overpass that coincides with the target location (green points). Assuming that OCO‐3 soundings will have a distribution similar to OCO‐2 soundings, it is evident that the prescribed s‐SAM distribution reflects the latitudinal spacing but overestimates the longitudinal spacing of simulated soundings. (SAM, snapshot area mapping; OCO, Orbiting Carbon Observatory).

To reduce the computation required for the evaluation process and focus the analysis on urban CO_2_ emissions, this work uses a domain smaller than a typical SAM. Figure 3 (top) depicts a full SAM domain (∼100 km × ∼80 km) with the smaller inner domains representing the areas used for this study. Dimensions are 48  × 41 km and 52 × 41 km for the inner domains of LA and SLC, respectively. To simplify the distribution, analyses, and discussions of soundings within each s‐SAM, their spacing was constrained to 0.019 ° × 0.019 ° (roughly 2.1 km), keeping the length scales in the longitudinal and latitudinal directions consistent. It was assumed that the swaths making up each SAM were adjacent. Included in Figure 4 is a depiction of the s‐SAM sounding distribution over SLC. Accompanying this distribution is an OCO‐2 overpass that coincides with the target location. Assuming that OCO‐3 soundings will have similar spacing to that of OCO‐2, it is clear that the intended s‐SAM distribution is reflective of the proper latitudinal spacing yet over estimates longitudinal spacing. Given the dimensions of inner domains and intersounding spacing, the domain over SLC accommodates 500 soundings. The domain over LA accommodates 460. Although it is expected that the SAMs provided by the OCO‐3 instrument may vary in orientation and/or coverage of target locations, it is assumed that they will largely cover the same area as the inner domains presented here.

As described by Eldering et al. (2019), OCO‐3’s SAM mode provides scanned geographic regions on the order of 100 × 100 km with a sounding spacing of roughly 1.6 × 2.2 km; furthermore, the orbit of the ISS allows SAMs to be collected at various times of day. To accommodate Eldering et al.’s specifications in the interpolation method’s evaluation, the s‐SAMs generated for this work are sampled from a 6 month period (January to June of 2017). Roughly 3 days were selected from each month. On each selected day, three s‐SAMs were generated, reflecting morning, noon, and afternoon times of day. Specific dates and times are included in Table 1. This sampling encapsulates the transition from winter months to summer months while the generation of three s‐SAMs on each day will reflect unique diurnal events that may be found at each location.

**Table 1:** *To Capture a Broad Array of Meteorological Characteristics in s‐SAMs, 15 days Were Selected Across 6 Months of 2017 (January to June)*

Month	Day	City	Times (UTC)
January	2, 12, 22	Los Angeles	16:00
February	1, 11, 21		19:00
March	3, 23		23:00
April	2, 12, 22	Salt Lake City	15:00
May	2, 12, 22		18:00
June	1		22:00

This subset of days is reflective of the winter‐to‐spring transition experienced at both evaluation locations. Further stratification is introduced by generating three s‐SAMs for each day. These s‐SAMs are generated at morning, noon, and evening local times.

In this work, only the effects of anthropogenic CO_2_ emissions are considered; therefore, ODIAC was used to represent the urban areas and their contribution to the ΔXCO_2_ found in each s‐SAM. ODIAC is reflective of both the difference in population between the two cities and their number of large industrial CO_2_ sources. In Figure 3 (bottom), ODIAC fluxes have been averaged from January to June of 2017 and are displayed in the context of each test location’s inner s‐SAM domain. Large CO_2_ fluxes (>50 μmol/m^2^/s) are indicated by red points. Although the spatial extent of the s‐SAMs is contained within the inner domains, their footprints were generated on the larger (*x*, *y*) grid (20° × 20° ). This includes any influences from the regions outside of the urban areas. The spatial extent of ODIAC used for ΔXCO_2_ calculations was identical to the (*x*, *y*) grid’s extent. A total of 90 s‐SAMs were generated by passing the sets of column receptors and appropriate times to the X‐STILT model. A footprint was generated for each receptor and then convolved with ODIAC (Equation 2). These ΔXCO_2_ values were treated as simulated OCO‐3 enhancement values.

### 2.3 Method of Interpolation

#### 2.3.1 Selecting Subsets

Using the PMA, OCO‐3 creates SAMs by conducting multiple adjacent transects over targets within a ∼2 min interval, providing 100s of spatially dense soundings. The mechanics and geometry of data collection may cause the inter‐sounding spacing to vary across these transects. Thus, soundings may not fall on a uniform grid. Furthermore, the spatial orientation of OCO‐3 provided SAMs may vary. These subtle variations between soundings do not permit grid indexing based strictly on latitude and longitude coordinates. Since the interpolation method requires spatial consistency across SAMs, soundings are indexed on a regular *m* × *n* grid, *S*. Here, *m* and *n* represent the number of rows and columns of soundings in a SAM. This two‐dimensional scheme allows each sounding (or missing sounding) to be referred to using indices *i* and *j* rather than latitude and longitude. This work does not attempt to anticipate the possible orientations of OCO‐3 generated SAMs nor the potential variations in intersounding distances; therefore, as noted in Section 2.2, all s‐SAMs are identically oriented. These consistencies simplify the mapping to *S*, analyses, and discussions of results.

The interpolation method is applied to subsets of *S*. Subsets are defined by constraining the indices of soundings such that subset *S*_*IJ*_ is given by:

(3) $$S_{IJ}=\left\{S_{ij}│(\alpha_{y}-1)(I-1)+1⋀i\leq I(\alpha_{y}-1)+1\wedge (\alpha_{x}-1)(J-1)+1\leq j\leq (\alpha_{x}-1)\cdot J+1\right\}.$$

**Figure 5:**
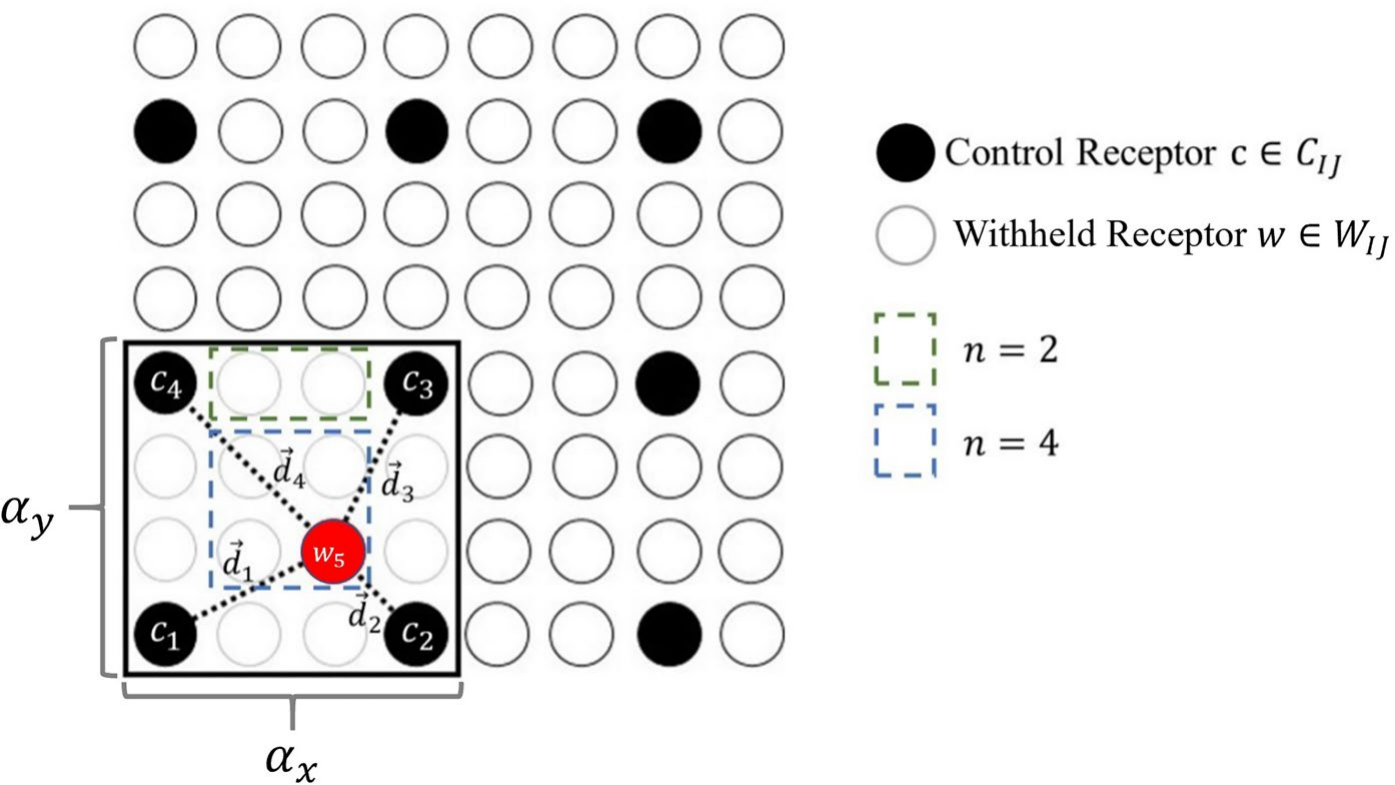
Depicted here is an analog to a full SAM (64 soundings). A subset is selected such that *α*_*x*_ = *α*_*y*_ = 4. In this scenario, a footprint for withheld receptor *w*_5_ is to be interpolated (red point). This requires each footprint generated for the *c*_*k*_s (black points) to be translated by ${\vec{d}}_{k}$ and averaged using an inverse weighting scheme. For any withheld receptors that fall directly between two control receptors (dashed green box) only the two closest control receptors are used in the interpolation method. All other interior receptors (dashed blue box) are constructed using all available control receptors. (SAM, snapshot area mapping).

Here, *s*_*ij*_ represents individual soundings. Sounding indices, *i* and *j*, are constrained by the indices of the subset of interest: *I* and *J*. The scaling parameter *α* reflects the extent of each subset such that *α*_*x*_ and *α*_*y*_ indicate the columns and rows of soundings in each *S*_*IJ*_. Figure 5 demonstrates the selection of subset *S*_1,1_ from a SAM analog. This subset (black rectangle) has four rows and four columns of soundings, thus *α*_*x*_ = *α*_*y*_ = 4. Soundings that are a part of this subset are thus defined as: *S*_1,1_ = {*s*_*ij*_ | 1 ≤ *i* ≤ 4⋀1 ≤ *j* ≤ 4}.

The interpolation method presented here requires preexisting X‐STILT footprints for soundings located at the corners of each subset. These soundings are indicated as black points in Figure 5. The minimum and maximum *i*/*j* index values in each subset are required to identify these soundings. These values are denoted as *i*_min,*IJ*_/*j*_min,*IJ*_ and *i*_max,*IJ*_/*j*_max,*IJ*_, respectively. The subset of soundings requiring X‐STILT generated footprints are defined as:

(4) $$C_{IJ}=\{s_{i_{\min,IJ},j_{\min,IJ}},s_{i_{\min,IJ},j_{\max,IJ}},s_{i_{\max,IJ},j_{\max,IJ}},s_{i_{\max,IJ},j_{\min,IJ}}\}.$$

The remainder of soundings within the interior of the subset *S*_*IJ*_ are withheld, not being passed to the X‐STILT model. Equation 5 below prescribes the construction of this repressed set.

(5) $$W_{IJ}=S_{IJ}-C_{IJ}.$$

In the construction of each *S*_*IJ*_, *C*_*IJ*_, and *W*_*IJ*_, it should be noted that only subsets where |*C*_*IJ*_| = 4 are considered. For the purposes of this work, requiring each *C*_*IJ*_ to contain four soundings ensures that only interpolated data is included and extrapolated data is excluded. The selection of the subset size in tandem with the dimensions of *S* may result in soundings near the periphery of the SAM/s‐SAM to not be assigned to a subset. These elements are also passed to the X‐STILT model and footprints are generated using the traditional method. An example of soundings that are not assigned to a subset is demonstrated by the uppermost row and rightmost column of the SAM analog presented in Figure 5.

#### 2.3.2 Applying the Interpolation Method to Subsets

The geolocations associated with the soundings in each *S*_*IJ*_ become column receptors for X‐STILT and the interpolation method. Using X‐STILT footprints associated with each control receptor location (Equation 4), a synthetic footprint is generated for each withheld receptor in *W*_*IJ*_. A spatial shift and inverse square weighting are applied to the X‐STILT footprints. This approach applies the highest weighting value to nearby footprints while reducing the influences of more distant footprints. Calculating the elements of an interpolated footprint, $\hat{f}\left(x_{i},y_{j},t_{0}\right)$, is achieved by the following:

(6) $$\hat{f}\left(x_{i},y_{j},t_{0}\right)={\left(\overset{n}{\underset{k=1}{\sum }}\frac{1}{1+|{\vec{d}}_{k}{|}^{2}}\right)}^{-1}\left(\overset{n}{\underset{k=1}{\sum }}\frac{1}{1+|{\vec{d}}_{k}{|}^{2}}\cdot f_{k}\left(x_{i}+\Delta \lambda_{k},y_{j}+\Delta \phi_{k},t_{0}\right)\right).$$

For a particular withheld receptor, *w* ∈ *W*_*IJ*_, its synthetic footprint is determined by taking the individual values making up control footprints from *C*_*IJ*_ receptors and averaging them according to Equation 6. The haversine distance between *w* and each *c*_*k*_ ∈ *C*_*IJ*_ is used as the weighting: ${\vec{d}}_{k}=\langle \Delta \lambda_{k},\Delta \phi_{k}\rangle$. The collection of synthetic footprint elements, $\hat{f}\left(x_{i},y_{j},t_{0}\right)$, constitutes a complete synthetic footprint for a repressed receptor such that $\hat{f}\left(x_{i},y_{j},t_{0}\right)\in \hat{F}\left(w\left(\lambda ,\phi ,t_{0}\right)\right)$.

Qualitatively, this method translates control footprints from *C*_*IJ*_ locations to the location of each withheld receptor (*W*_*IJ*_), performing a point‐wise averaging to generate synthetic footprints. Figure 5 depicts the construction of a subset using a SAM analog consisting of 64 soundings. Inside the subset identified by the black box, a synthetic footprint is generated for a withheld receptor (red). The distances, ${\vec{d}}_{k}$, are used as weights in Equation 6 and determine where control footprints are to be translated. A subtlety is introduced when Equation 6 is applied. If the withheld receptor of interest shares a longitude or latitude value with two of the control receptors, then only the two closest control footprints will be used in the interpolation. This is indicated in Figure 5 by the dashed green box. Here, only control footprints from *c*_3_ and *c*_4_ are used. For interior withheld receptors (dashed blue box), all control footprints are used.

### 2.4 Metrics for the Comparison of Footprints

Serving as controls for each comparison in this study, the series of s‐SAMs described in Section 2.2 was generated using X‐STILT. Each control s‐SAM had an accompanying series of s‐SAMs constructed by using the interpolation scheme, increasing the subset extent to *α*_*x*_ = *α*_*y*_ = 3, 4, 5, 6, and 7. These extent parameters corresponded to length scales of 4 , 6, 8, 10, and 12 km, respectively. To evaluate the interpolation scheme’s effectiveness, each interpolated s‐SAM was compared to its corresponding control. Comparisons were drawn between (1) each column‐based receptor value with and without convolution with ODIAC (without convolution, *F*_tot_ = *∑*_*ij*_ *f* (*x*_*i*_, *y*_*j*_, *t*_0_); with convolution, see Equation 2), and (2) each spatial distribution of control and interpolated footprint elements. In the first case, changes in the Pearson correlation coefficient (*r*), root mean squared error (RMSE), and mean bias error (MBE) as a function of subset extent were investigated. For footprint‐to‐footprint comparisons, two aspects were considered. A threat score (TS), commonly used in forecast verification, was computed to quantify the spatial agreement between an interpolated footprint and its associated control footprint (Jolliffe & Stephenson, 2003). Conversely, a spatially weighted mean absolute error (WMAE) calculation was used to quantify the agreement between corresponding footprint element values. Specific methodology regarding the TS and WMAE metrics is discussed in Section A1.

### 2.5 Comparison of XCO_2_ Errors

Generally, errors in ΔXCO_2_ are minimized when differences in interpolated footprints are reduced yet scenarios exist where subtle disagreements within an interpolated footprint can be magnified by the distribution of fluxes in Φ(*x*_*i*_, *y*_*j*_). Errors associated with interpolated ΔXCO_2_ values can be addressed in the context of OCO‐2,3 error; however, OCO‐3 SAMs were not fully released at the time of writing. Since these two observational platforms were constructed with identical instrumentation, it was assumed that OCO‐3’s error characteristics will be similar to those of OCO‐2. The notable difference between these two instruments is in the spatial coverage that OCO‐2 provides. In standard operating mode, OCO‐2 observes a narrow transect of the atmosphere (Figure 4). For this work, OCO‐2 transects were selected such that their swath width was within the s‐SAM domain at each testing site. Errors reported with quality assured soundings were used to characterize the distribution of observational errors.

Every XCO_2_ sounding (in ppm) in an OCO‐2 transect is reported with a error value such that XCO_2,*τ*_ ± *δ*XCO_2,*τ*_, were *τ* indicates a particular sounding in the transect. The individual error values, *ɛ*, within these distributions were defined as:

(7) $$\varepsilon_{\tau }={XCO}_{2,\tau }-\hat{{XCO}_{2,\tau }}.$$

$\hat{{XCO}_{2,\tau }}$ is randomly selected from a uniform distribution, *U*(−*δ*XCO_2,*τ*_, *δ*XCO_2,*τ*_). Five thousand values of *ɛ* were generated for each site. Constructing errors with this method provided customized distributions at both testing locations whose characteristics were constrained by realistic observations. The transects used are specified in Table 2.

**Table 2:** *A List of Available OCO‐2 Transects are Presented Here*

Location	M/D/Y	Time (UTC)	Soundings
Los Angeles	September 12, 2014	21:12	249
Los Angeles	October 07, 2014	21:05	1191*
Salt Lake City	November 21, 2014	20:35	125
Salt Lake City	December 23, 2014	20:35	116
Salt Lake City	October 07, 2015	20:35	119
Salt Lake City	December 10, 2017	20:18	55
Salt Lake City	January 16, 2018	20:35	124

The number of soundings in each transect is given in the fourth column after quality filtering has been applied. Soundings provided by the second transect associated with the Los Angeles test site, indicated by (*), were taken in OCO‐2’s “target” mode.

### 2.6 Large Point Source Detection Algorithm

When applying this interpolation scheme, the magnitude and number of induced errors can be driven by local meteorology, topography, and large point sources. Significant topography can reduce atmospheric transport and allow for the buildup of XCO_2_ without a large surface‐based CO_2_ source acting as a driver. These features are coupled with meteorological characteristics and are variable in both space and time; Conversely, large point sources of CO_2_ are better anticipated. Although the magnitude and dispersion of XCO_2_ enhancements may vary due to meteorology, variations in point source plumes are constrained by their locations and consistent output. Large point sources (LPSs) contribute a significant fraction of national CO_2_ emission totals and form localized XCO_2_ enhancements (Nassar et al., 2017; Singer et al., 2014).

A key feature of this interpolation method that is affected by large point sources lies in the hypernear‐field (HNF) of synthetic footprints. This area typically covers length scales of 1–10 km and timescales of 0.1–1 hours. In this area, surface fluxes are weakly diluted and thus more strongly influence the receptor (Fasoli et al., 2018). This causes a dense area of particles near the receptor that tapers off over time and space. As nearby footprints are selected for the interpolation process, the lack of mixing in their concentrated HNFs causes any interfootprint variations to be averaged into a single, smoothed footprint with a larger spatial HNF distribution. Thus, a smoothed synthetic footprint may interact with a nearby point source that would have been missed by an X‐STILT generated footprint at the same location.

**Figure 6:**
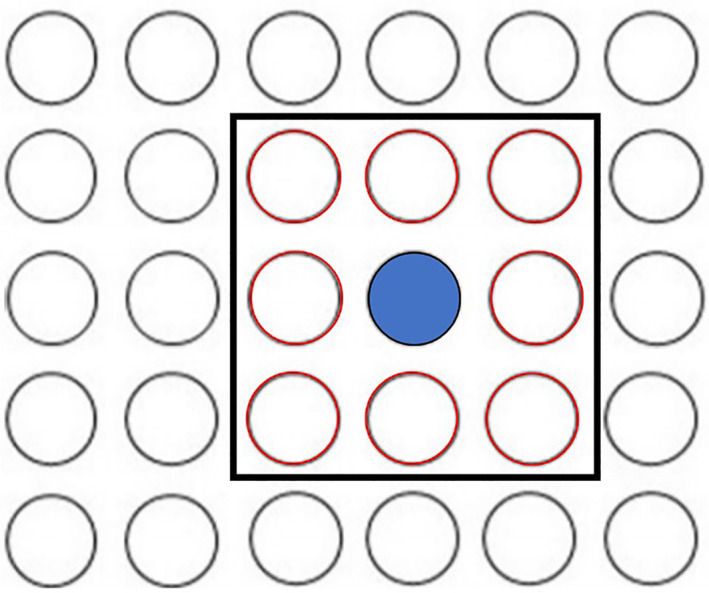
Here, a Moore neighborhood is constructed for a particular sounding in a domain (blue). The 3×3 neighborhood contains this sounding and its adjacent soundings (red).

In this work, the potential errors associated with large point sources are addressed by implementing a large point source detection algorithm (LPS‐DA). This algorithm is tuned to detect XCO_2_ enhancement characteristics within an s‐SAM that are associated with large point sources. Any soundings identified by this algorithm were not interpolated and instead passed to the X‐STILT model. This detection process constructed a Moore neighborhood around each sounding of each s‐SAM, with the extent encapsulating immediately adjacent soundings. The XCO_2_ value of each central sounding was compared to the average value of the adjacent soundings. If the difference was greater than 1ppm then no soundings in the Moore neighborhood were interpolated. An example of a Moore neighborhood can be found in Figure 6.

### 2.7 Investigating Effects of Meteorological Resolution

The final parameter investigated was the grid spacing of the meteorological fields. Data from the Weather Research and Forecasting (WRF) model (Nehrkorn et al., 2010) were available for September and October of 2015 for SLC at three different spatial resolutions: 1.33, 4, and 12 km (Kunik et al., 2019; Lin et al., 2017; Mallia et al., 2015). Applying the same methodology as described in Section 2.2, 5 days were selected and s‐SAMs were constructed at three different times per day. The specific days and times are included in Table 3. Using WRF, it is possible to ensure that the only variable being changed is the resolution, keeping all other model physics the same. Errors were calculated for all interpolated XCO_2_ enhancements (ΔXCO_2,interpolated _− ΔXCO_2,control_). For each subset length scale, differences in the groups of errors generated by the 1.33, 4, and 12 km WRF data were investigated using a Kruskal‐Wallis H test. This test was used to detect a statistically significant difference between the means of errors associated with the 1.33, 4, and 12 km WRF resolutions. A discussion on the Kruskal‐Wallis H test is provided in Section A2. Since WRF is used solely as a means to modify meteorological input resolution, comparisons between WRF and HRRR are beyond the scope of this paper.

**Table 3:** *A Small Sample of s‐SAMs was Generated Using the Weather Research and Forecasting (WRF) Model*

Month	Day	Times (UTC)	Grid spacing (km)
September	2, 15, 30	15:00	1.33
October	12, 30	18:00	4
		22:00	12

These data were available from previous analyses performed for the year 2015 and therefore was only available for Salt Lake City. Each s‐SAM was created using three different WRF resolutions.

## 3 Results

### 3.1 Comparison of Summed Footprint Values

**Figure 7:**
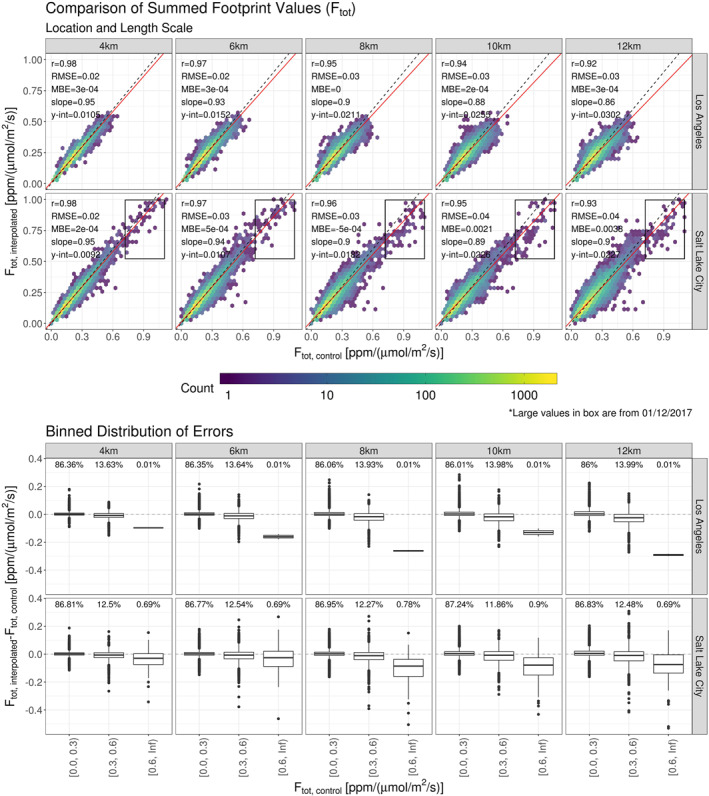
(Top) Stratified by location and subset length scale, summed values of X‐STILT generated footprints are compared to their interpolated counterparts. Each panel contains column‐averaged receptors from s‐SAMs representative of 15 days. Three times were sampled per day to incorporate diurnal features. These values are compared independently of their geographic location. Density of compared values are represented by the color gradient. The 1:1 ratio line is indicated by the black dashed line and the line of best fit is represented by the red line. Statistics from each fit are supplied in the top left corner of each panel. The anomalous values indicated in the bottom row are from January 12, 2017. (bottom) Binned by the corresponding control value, summed values of X‐STILT generated footprints are compared to their interpolated counterparts. Each panel contains a box plot of binned errors. Above each distribution is the percentage of values within the bin. (X‐STILT, Stochastic Time‐Inverted Lagrangian Transport; SAM, snapshot area mapping).

Typically, X‐STILT footprints are used in tandem with an emission inventory to determine a priori column‐averaged CO_2_ enhancements (Equation 2); however, the evaluation of this interpolation method first considered only the summed values of footprint elements to remove any potential influences from ODIAC. In Figure 7, summed footprint values (*F*_tot_ = *∑*_*ij*_ *f* (*x*_*i*_, *y*_*j*_, *t*_0_)) are compared to their interpolated counterparts, stratified only by subset length scales. Comparisons of all values revealed strong correlations across length scales with *r* > 0.9 in all cases. The correlation coefficient was inversely proportional to the subset length scale which reflected the loss of accuracy when larger subsets were used for interpolation. This loss was also reflected in RMSE values. From a 4 km to a 12 km length scale, RMSE increased by 50% (0.2–0.3) and 100% (0.2–0.4) at LA and SLC, respectively.

Between both testing locations, 99.8% of all *F*_tot, control_ values fell below 0.7 ppm/μmol/m^2^/s. The remaining 0.2%, indicated in Figure 7, were all attributed to a single day (January 12, 2017) at the Salt Lake City location. Spatially, these particular receptor locations were constrained to the area over the Oquirrh Mountains to the west of the city on a day where higher elevations experienced little atmospheric transport. As the subset length scale was increased these interpolated soundings began to underestimate larger modeled values. This demonstrates that uncertainties in the interpolated results are sensitive to large *F*_tot_ values and complex terrain. These uncertainties decreased when smaller subset length scales were used.

An ordinary least squares regression was performed on the comparisons in Figure 7 (top) and the coefficients were included in each panel. Although the linear model assembled here is not used, the calculated values of slope and *y*‐intercept offer insights into the interpolation method’s ability to reproduce *F*_tot_ values. At both locations, slope values decreased as the subset length scale increased; conversely, *y*‐intercept values increased as the length scale increased. Slopes lying below the 1:1 line reflected a tendency to underestimate *F*_tot_ values due to averaging in the HNF. The trend of this parameter indicated that HNF smoothing effects increased as the subset length scale increased.

Although the systematic error suggested by each *y*‐intercept was relatively large, the MBE was driven by the majority of *F*_tot_ values lying between 0.0 and 0.3 ppm/μmol/m^2^/s. The density of values in this range is shown in Figure 7 by the high count of the weighted scatter plot (top). Additionally, the percentage of points within this range is provided above the corresponding box plots (bin [0.0, 0.3); bottom). The amount of values within this range was consistently ≥86% for both locations and all length scales. Coefficients from the linear regression and accompanying boxplots demonstrate that this interpolation scheme has a tendency to overestimate small *F*_tot_ values and more significantly underestimate larger *F*_tot_ values. For the 10  and 12 km subset length scales, MBE values at the SLC test location were significantly higher than MBE values for length scales at LA. This demonstrated the geographic sensitivity of the method; however, given that at least 86% of values lie below 0.3 ppm/μmol/m^2^/s for both locations, it is likely that any interpolated footprints in future work will have a bias on the order of 10^−4^ ppm/μmol/m^2^/s.

### 3.2 Spatial Comparison of Summed Footprint Values

**Figure 8:**
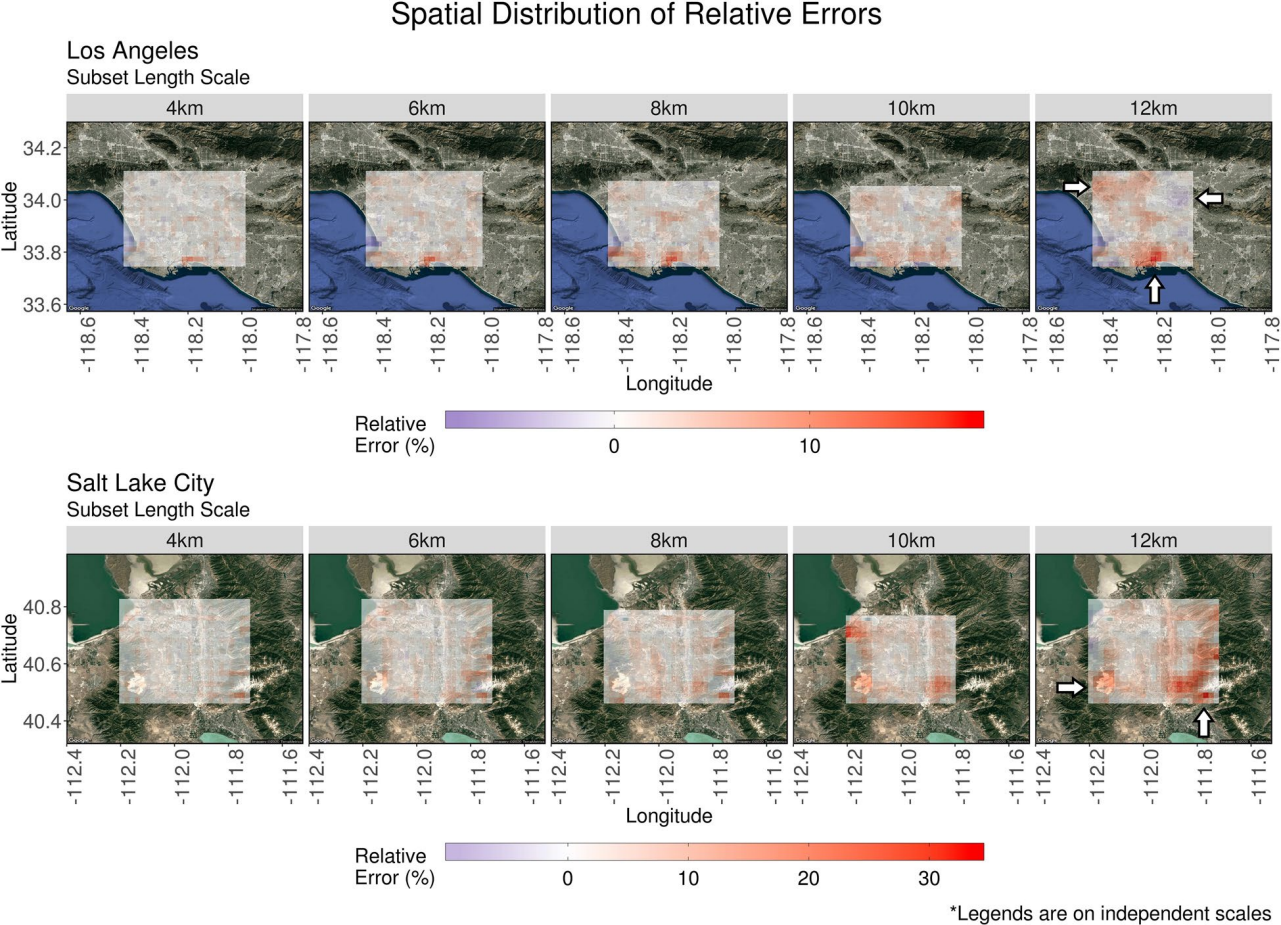
Stratified by location and subset size, relative errors of interpolated summed footprint values were spatially averaged. Each panel contains column‐averaged receptors from s‐SAMs representative of 15 days. Three times were sampled per day to incorporate diurnal features. Noninterpolated values from X‐STILT generated footprints are included with a relative error of 0%. These footprints were required for the interpolation process and therefore are not compared to other values. At large length scales, error clusters begin do develop. These clusters are indicated by white arrows. (The legends accompanying these plots are on independent scales.) (X‐STILT, Stochastic Time‐Inverted Lagrangian Transport. SAM, snapshot area mapping).

In Figure 7 (Top), comparisons of *F*_tot, interpolated_ were considered independently of their locations whereas Figure 8 presents the same errors spatially distributed across the s‐SAM domains. To evaluate how errors are distributed in space, all s‐SAMs at each location were stratified by their subset length scales and averaged. At small length scales, errors are smaller and no clusters exist. As the length scales increase, clusters of larger error appear.

Over Los Angeles, three clusters of large error exist: an area to the south that is on the land‐sea border, an area to the northwest at the base of a small mountain range, and an area near a small mountain range to the northeast. It is assumed that column‐based receptors generated over bodies of water are propagated with different model physics than those generated over land. These differences were exaggerated at larger length scales as more distant footprints were used for interpolation. In Figure 8 (top), the large positive error indicates that the interpolated footprint was overestimating the correct summed value. In addition to proximity to bodies of water, close proximity to mountains can influence near‐surface winds. As the subset length scale increased, the interpolation increasingly overestimated values around the base of the northwestern mountain range. Conversely, there was an area of underestimation that appeared around the base of the smaller northeastern mountain range. Of these three areas of higher/lower error, the receptors at the land‐sea border were consistently the largest value and highest concentrated. At SLC, an increasing tendency of overestimation appeared to the southeast along the Traverse Mountains, stretching northward into the Wasatch Mountains. At the larger length scales, overestimates occurred along the left side of the s‐SAM domain over the Oquirrh Mountains.

Viewed through the element‐wise summation of footprints, the method’s ability to interpolate these values was broadly demonstrated. Comparisons showed that small *F*_tot_ values were overestimated while larger, less‐frequent values were underestimated by this method. The amount of under/over‐estimation was dependent on the subset length scale used in the interpolation process. The method’s sensitivity to significant geographic features was also evident as relative errors increased near land‐sea borders and mountainous terrain. Although similar influences exist at both test locations, error values are not comparable in magnitude. This is due to the unique meteorological and topographical characteristics found at each location. LA experienced a maximum relative error of 18.2% along the land‐sea border when the largest subset length scale (12 km) was used. Similarly, the testing domain across SLC contained a maximum relative error of 33.4% atop the Traverse Mountains while using the same length scale. Regardless of errors associated with geographical features, values are relatively small (<10%) when interpolating across smaller length scales.

### 3.3 Footprint‐To‐Footprint Comparison

**Figure 9:**
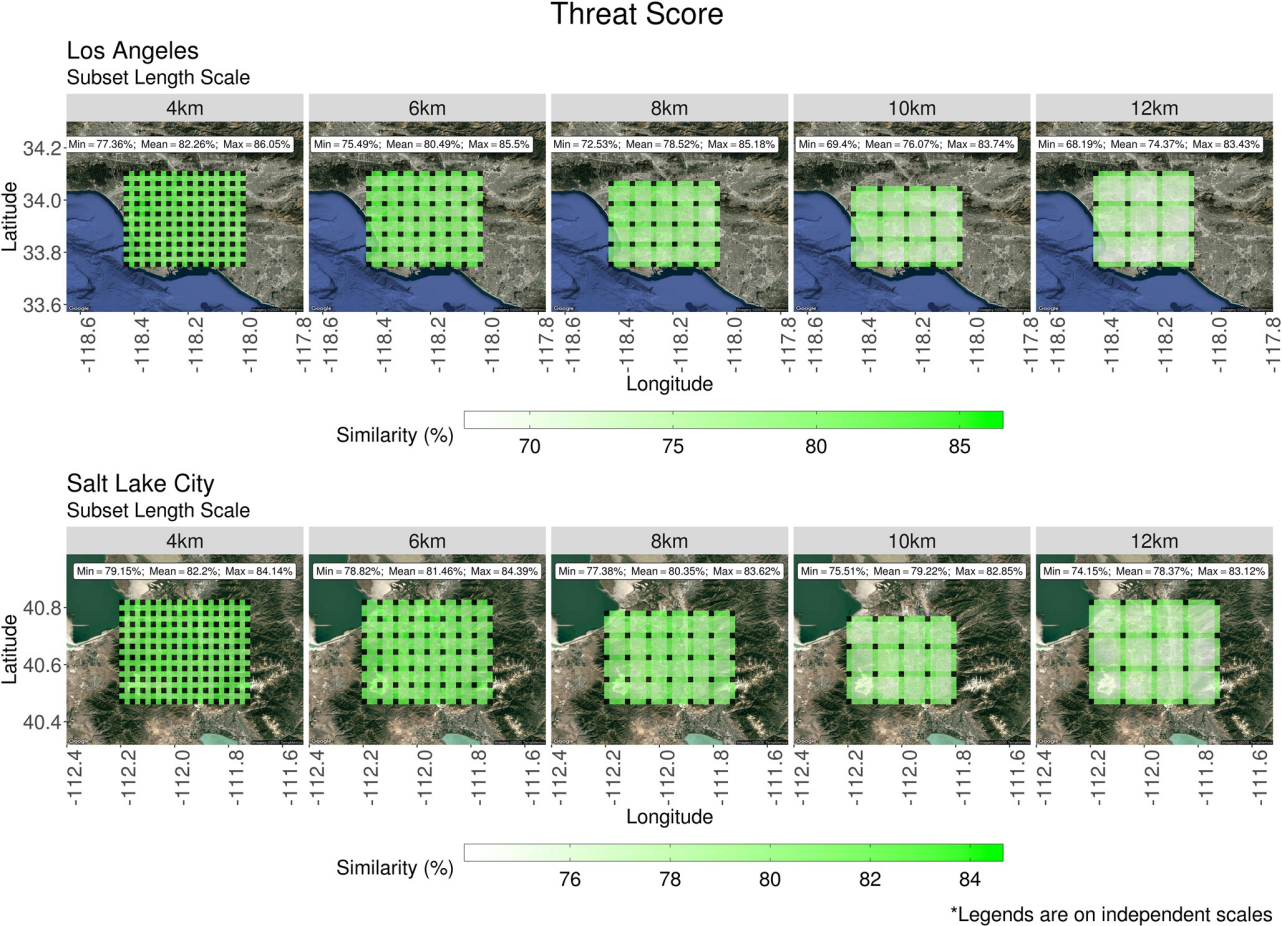
Stratified by location and subset size, threat scores of interpolated summed footprints were spatially averaged. Each panel contains column‐averaged receptors from s‐SAMs representative of 15 days. Three times were sampled per day to incorporate diurnal features. Locations of control receptors are represented by black tiles. Footprints from these receptors were required for the interpolation process and therefore are not compared to other values. The minimum, mean, and maximum values are reported for each panel. (The legends accompanying these plots are on independent scales.) (SAM, snapshot area mapping).

**Figure 10:**
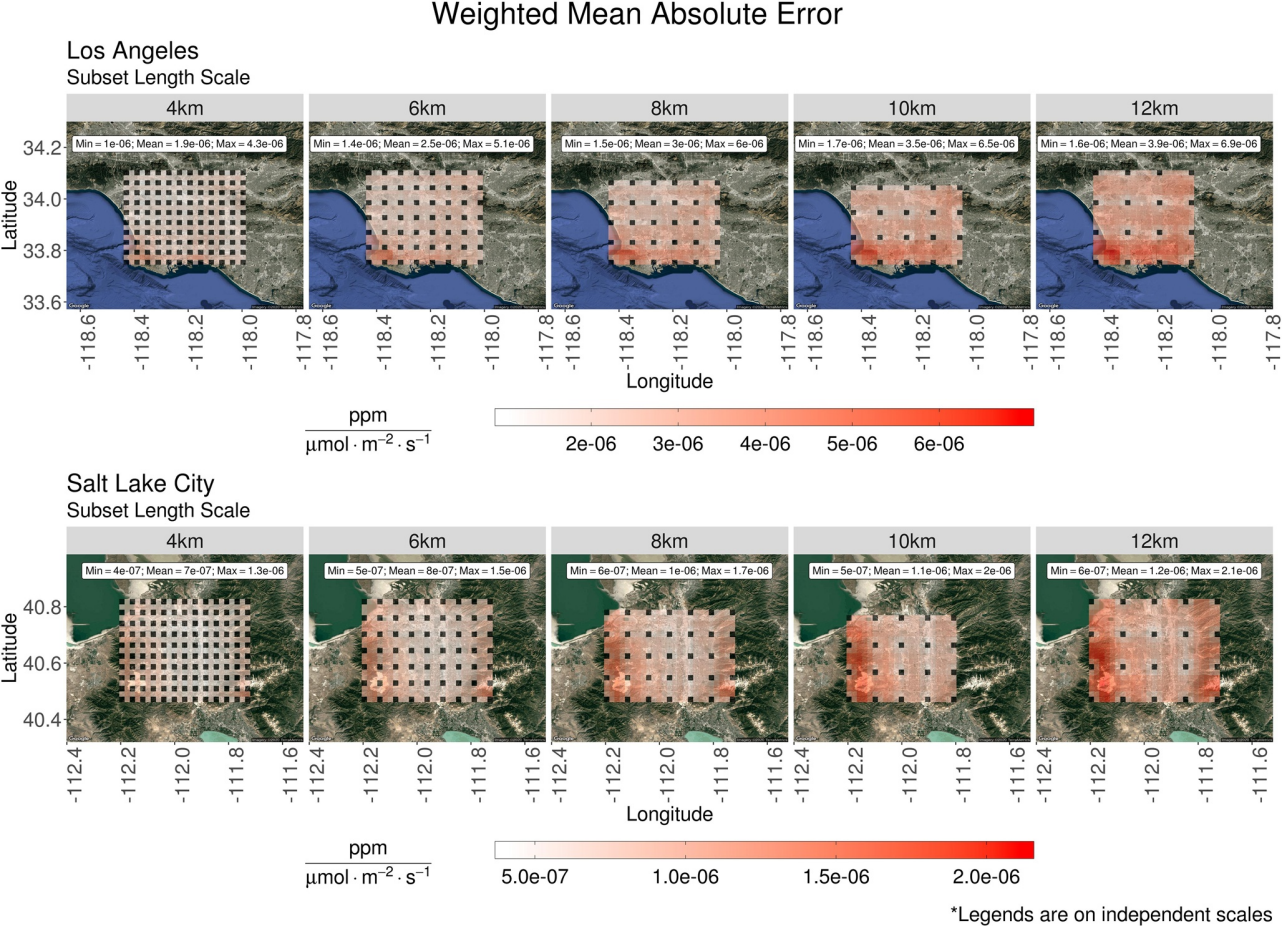
Stratified by location and subset size, the weighted mean absolute error of interpolated summed footprints was spatially averaged. Each panel contains column‐averaged receptors from s‐SAMs representative of 15 days. Three times were sampled per day to incorporate diurnal features. Locations of control receptors are represented by black tiles. Footprints from these receptors were required for the interpolation process and therefore are not compared to other values. The minimum, mean, and maximum values are reported for each panel. (The legends accompanying these plots are on independent scales.) (SAM, snapshot area mapping).

Error clustering, found in Figure 8, was investigated with methods described in Section 2.4. First, the threat score (TS) from Equation A3 was applied to all interpolated footprints. Spatial characteristics of interpolated footprints were compared to the control footprint associated with the same receptor location. Results of this comparison were included in Figure 9. In addition to the comparison of spatial characteristics, the interpolation method’s ability to reproduce values was also considered. The weighted mean absolute error (WMAE) calculation of Equation A4 was applied to characterize the difference between control footprint element values and their interpolated counterparts. The results of this comparison are presented in Figure 10.

Stratified only by the length scale used to generate them, TS values were assigned to their respective receptor locations and spatially averaged. The mean of these averaged threat scores ($\bar{TS}$) was greater than 70% in all panels of Figure 9. Smaller scales (4  and 6 km) produced $\bar{TS}$ values that were uniform across the s‐SAM domain. Among these smaller scales, the largest range in values (*TS*_*max*_ − *TS*_*min*_) was 10% and attributed to the 6 km length scale at the LA location. Beyond the 6 km subset length scale, systematic differences began to appear. At these larger subsets, it was clear that interior $\bar{TS}$ values disagreed considerably with $\bar{TS}$ values along the periphery of subsets. As the subset length scale increased, so did the variation among control footprints used in the interpolation process. Using only two control footprints to synthesize a third footprint introduces less variation than using four; therefore, synthetic footprints along the periphery of each subset had better spatial agreement to control footprints than those requiring four X‐STILT generated footprints.

Overall, the spatial accuracy of synthetic footprints generated at LA was more sensitive to the subset extent when compared to the SLC location. At the 12 km length scale, the minimum threat score value at the LA location (*TS*_*min*_ = 68.19%) corresponded to an interior receptor whereas the maximum value (*TS*_*max*_ = 83.43%) corresponded to a receptor on the periphery of a subset. While this location had a 15% difference, the 12 km length scale at SLC had a difference of 9%. Across the range of length scales, the values of $\bar{TS}$ at LA experienced roughly twice the decrease relative to SLC; Any geographic contributions to spatial mismatch were dominated by the variation in meteorology used to generate each footprint.

Spatial agreement between synthetic and control footprints was driven primarily by variations in the meteorology used in their construction; however, when comparing the values of footprint elements, influences of geographic features were evident. Applying Equation A4 to the elements of interpolated and control footprints, the WMAE values associated with each receptor were spatially averaged ($\bar{WMAE}$). The results of this averaging are presented in Figure 10. In the southwest corner of the s‐SAM domain over LA, a spatial cluster of higher $\bar{WMAE}$ values was present across all subset length scales. As the length scale increased, the errors in this region intensified and became more evident along the land‐sea border to the south. This region of higher $\bar{WMAE}$ occurs in the same location as the area of higher *F*_tot_ error in Figure 8 (top). The mountain ranges surrounding SLC demonstrate a similar effect on the $\bar{WMAE}$ of synthetic footprint elements. At the smallest possible implemented length scale (4 km), subtle clustering exists along the east and west edges of the s‐SAM domain. These errors increased as the subset size increased.

Both Figures 9 and 10 indicate the locations of column‐based receptors whose footprints were used in the generation of synthetic footprints. Since TS values are not applicable for these receptor locations, they are represented by black tiles in Figure 9. Likewise, these locations were represented in Figure 10 as locations with no error. At the largest length scale (12 km), two footprints from control receptors along the Los Angeles land‐sea border were averaged with two footprints that were 12 km inland. This difference was likely driving the clustered $\bar{WMAE}$ values in this region. Over the mountain ranges in the SLC domain, the 12 km length scale required the footprints from mountaintop receptors to be averaged with footprints generated 12 km away in the Salt Lake Valley. The difference in elevation across this large subset was the likely driver of the errors found along the Oquirrh and Wasatch mountain ranges. Given the characteristics of the $\bar{TS}$ and $\bar{WMAE}$ values in Figures 9 and 10, it appears that meteorological influences specific to the domain location introduce a uniform error into synthetic footprints while geographic features introduced localized errors.

### 3.4 ΔXCO_2_ Comparison to OCO‐2

**Figure 11:**
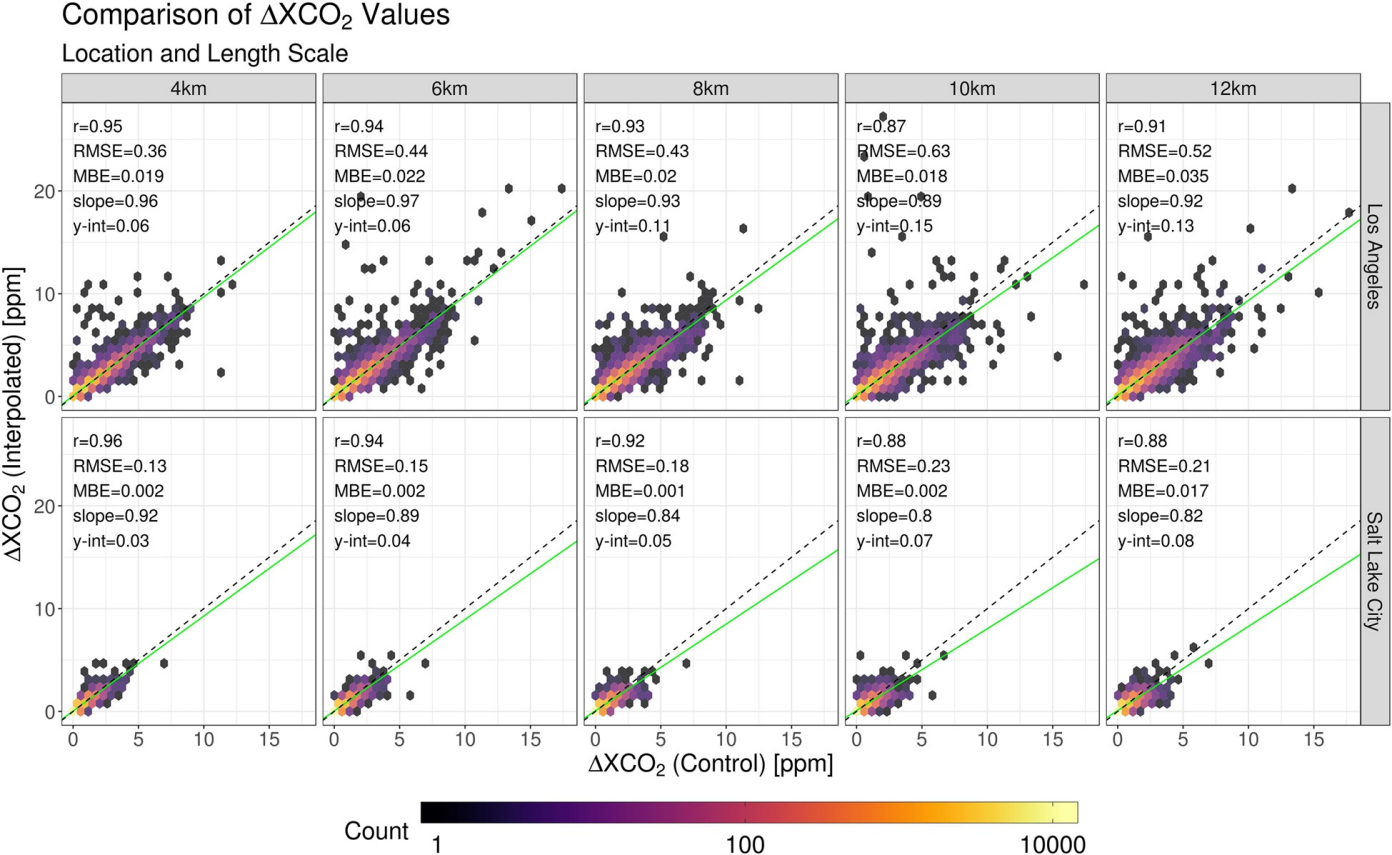
(Top) Stratified by location and subset length scale, s‐SAM ΔXCO_2_ values are compared to their interpolated counterparts. Each panel contains column‐averaged receptors from s‐SAMs representative of 15 days. Three times were sampled per day to incorporate diurnal features. These values are compared independently of their geographic location. The density of points within the comparisons are represented by the color gradient. The 1:1 ratio line is indicated by the black dashed line and the line of best‐fit is represented by the green line. Statistics from each fit are supplied in the top left corner of each panel. (SAM, snapshot area mapping).

Each footprint was convolved with the ODIAC emission inventory. Considering interpolation errors in “XCO_2_ space” provided an intuitive context for interpreting results and a means of selecting an upper threshold for appropriate subset length scales. Errors are presented in Figure 11 with noticeably different ranges between testing locations. Control values associated with SLC were predominantly constrained between 0 and 5 ppm whereas many values associated with LA were above this range. The difference in these ranges was reflective of the larger emissions and dense collection of large point sources in the Los Angeles metropolitan region; furthermore, RMSE and MBE values at the Los Angeles location were larger across all length scales than values found in the Salt Lake City area. RMSE values in LA were 100–200% greater than the associated SLC values. MBE values differed by an order of magnitude. Across both locations, underestimation by the interpolation method was still present. Slope values reported from linear least squares regression decreased as subset length scales increased. This trend was likely propagated by the mechanisms responsible for the systematic underestimation of larger *F*_tot, interpolated_ values (described in Section 3.1).

A collection of simulated OCO‐2 errors is presented with s‐SAM errors in Table 4. Although the spread of simulated OCO‐2 errors was constrained between ±1 ppm at both locations, it is possible for these errors to be larger (Connor et al., 2016). Therefore, s‐SAM errors are investigated using two different thresholds: (1) the percentage of values lying outside the range of simulated OCO‐2 error and (2) the percentage of values lying outside of the ±1 ppm range. Of the two testing locations, the distribution of s‐SAM errors at LA had a larger spread than values at SLC. At the LA location, s‐SAM errors associated with the smallest possible length scale (4 km) had a standard deviation of SD = 0.36ppm. This was larger than the SD of the OCO‐2 error (SD = 0.28 ppm). Like LA, the SD of s‐SAM errors for the SLC location increased as the length scale increased; however, the SD of s‐SAM errors for SLC was less than the SD of associated OCO‐2 error (<0.42 ppm) across all subset length scales. LA featured the largest min/max range across all length scales with the maximum range being 36.43 ppm (10 km length scale). Roughly 12% of the s‐SAM errors within this range were outside of the OCO‐2 error range with 3.5% lying outside of the ±1 ppm range. Unlike the LA location, the 10 km length scale at SLC had an associated s‐SAM error range of 9.53 ppm and <1% of the values were outside of the ±1 ppm range.

**Table 4:** *A Summary of Errors are Presented Here*

Location	Stat (in ppm)	OCO‐2 error	4 km	6 km	8 km	10 km	12 km
Los Angeles	Min	−0.48	−8.71	−5.47	−9.86	−11.25	−5.87
	Mean	0.00	0.02	0.02	0.02	0.02	0.03
	Max	0.48	9.53	17.59	9.92	25.18	13.1
	SD	0.28	0.36	0.44	0.43	0.63	0.52
	O.R. OCO‐2		4.51%	6.54%	9.96%	11.86%	14.42%
	O.R. 1 ppm		1.32%	1.7%	2.62%	3.47%	3.99%
Salt Lake City	Min	−0.72	−2.49	−4.62	−2.83	−4.38	−2.84
	Mean	0.01	0.00	0.00	0.00	0.00	0.02
	Max	0.72	3.03	2.99	3.19	5.15	2.35
	SD	0.41	0.13	0.15	0.18	0.23	0.21
	O.R. OCO‐2		0.39%	0.65%	0.96%	1.82%	1.73%
	O.R. 1 ppm		0.14%	0.23%	0.38%	0.81%	0.67%

The minimum (Min), maximum (Max), mean, and standard deviation (S.D.) are listed in ppm. The percentage of values lying outside of each location’s corresponding OCO‐2 error (O.R. OCO‐2) and the ±1 ppm range (O.R. 1 ppm) are also listed for each subset length scale.

At both locations, the SD increased with the subset length scale; conversely, the range of errors was not strongly correlated with the length scale but was driven predominantly by a few anomalous values created from large point sources and subset geometry. Across all length scales, SD values at the LA location were at least a factor of two greater than their SLC counterparts. These results demonstrate the interpolation scheme’s sensitivity to geographic location. The presence of both natural topography and anthropogenic CO_2_ emissions drive interpolation error. An additional constraint that was considered was the accuracy of the instrument collecting atmospheric soundings. Coarse measurements with large error will allow for the selection of larger subset length scales when the interpolation is applied. Overall error will be dominated by the instrument; however, as resolution/accuracy of the instrument is increased, the interpolation error must be further constrained to be smaller or equal to the instrument error. In the two locations presented in this work, usable length scales at LA were more restricted by instrument error. The s‐SAM errors at this location had a large spread while the available OCO‐2 error was small. Usable length scales at the SLC location were less constrained as there was less spread in s‐SAM errors yet larger spread in OCO‐2 error.

### 3.5 Applying the Large Point Source Detection Algorithm

**Figure 12:**
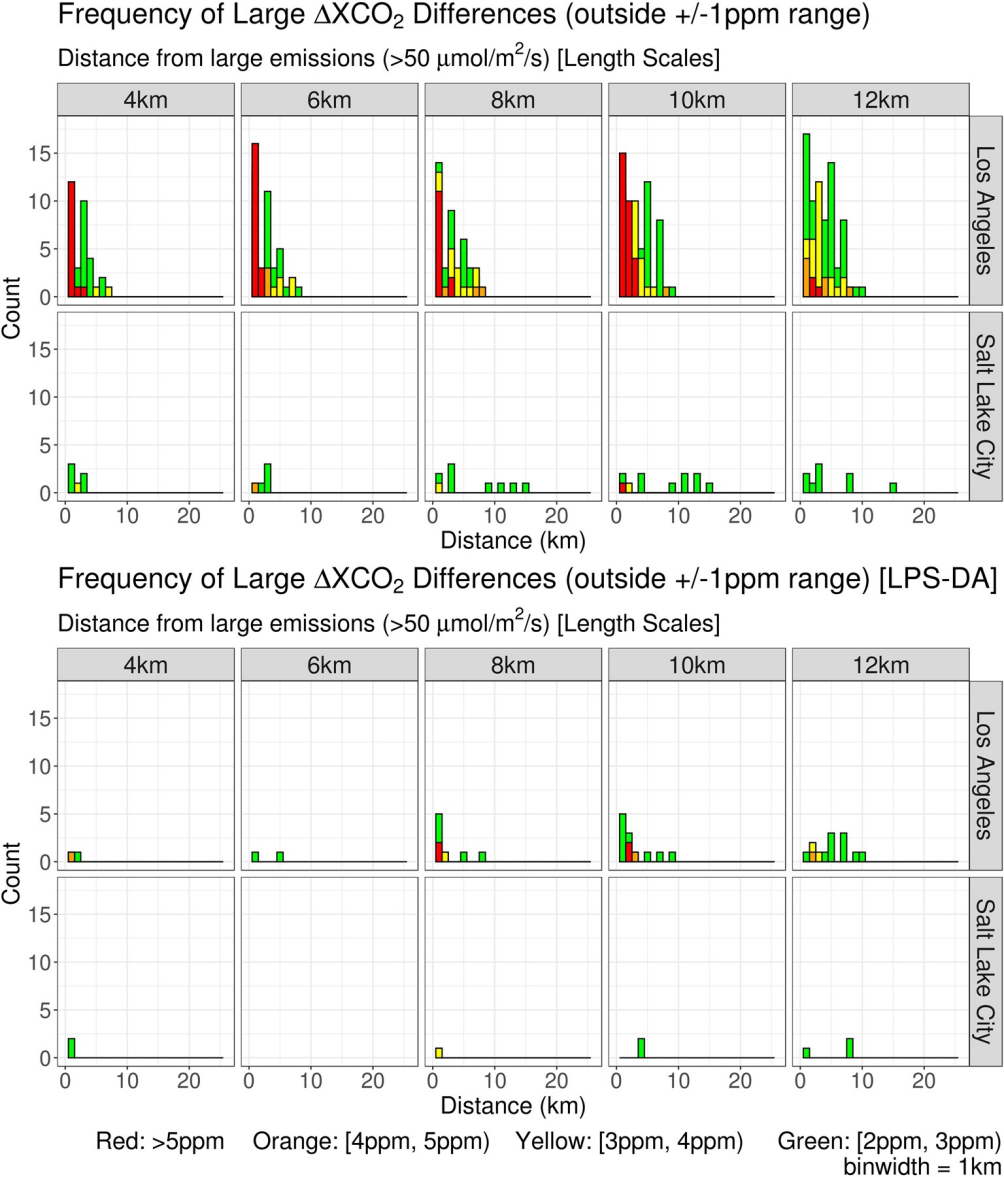
(Top) Stratified by location and subset length scale, s‐SAM ΔXCO_2_ errors outside the ±1 ppm range are counted and binned according to their distance from large ODIAC CO_2_ emission locations (defined as >50 μmol/m^2^/s). Each panel contains column‐averaged receptors from s‐SAMs representative of 15 days. Three times were sampled per day to incorporate diurnal features. Further binning is applied to the magnitude of the errors (in ppm). (bottom) Data presented in the bottom panel were collected and binned using the same methodology from above; however, a large point source detection algorithm was applied to reduce the number and magnitude of errors. (SAM, snapshot area mapping; ODIAC, Open‐source Data Inventory for Anthropogenic CO_2_).

The large point source detection algorithm (LPS‐DA) implemented in this work was selected to address large point sources (LPSs) and their influence on interpolated ΔXCO_2_ values. LPSs are included in ODIAC but, as with any emission inventory, errors in their locations may exist (Hogue et al., 2016, 2017; Hutchins et al., 2016; Oda et al., 2019); therefore, rather than constructing a detection method that relied on potentially incorrect location data from emission inventories, the Moore neighborhood approach (Section 2.6) was guided solely by the characteristics of s‐SAM values, anticipating source‐induced signals within them. When an assumed signal was detected, its distance from the nearest LPS was recorded (LPSs were defined as any ODIAC emission > 50 μmol/m^2^/s of CO_2_). The magnitude and frequency of error values were then binned by their respective distances to LPS values in ODIAC. These relationships are presented in Figure 12. It is important to note that OCO‐3 soundings were not used in this proximity‐based evaluation. Using ODIAC to generate s‐SAMs allowed LPS locations within the inventory to be treated as “truth.”

Although LPS signals were “assumed” in each s‐SAM, Figure 12 demonstrates a clear trend between large ΔXCO_2_ discrepancies and their proximity to LPSs in ODIAC. The distributions in the top panel of this figure represent the frequency of large errors and their distance from the nearest LPS. Not only did anomalous errors outside the ±1 ppm range occur more frequently near LPS locations, the magnitude of the error also decayed as distance increased. For LA, all errors >5 ppm occurred within the HNF of a LPS. The number of anomalous errors at SLC was smaller due to the low number of LPSs identified within the Salt Lake Valley. Furthermore, as the subset length scale increased at both locations, the number of anomalous errors near LPSs also increased. Figure 12 also presents anomalous errors after the LPS‐DA was applied (bottom panel). This reduced the magnitude and number of errors in the HNF of LPS locations. Most notable was the complete removal of ±1 ppm HNF errors in the 6 km interpolated s‐SAMs over SLC. After the application of the LPS‐DA, the magnitude and number of errors still increased with length scale; however, the number of anomalous errors was reduced across all length scales and the spatial distribution of (2 ppm, 3 ppm) errors was constrained to the HNF of LPS locations.

**Figure 13:**
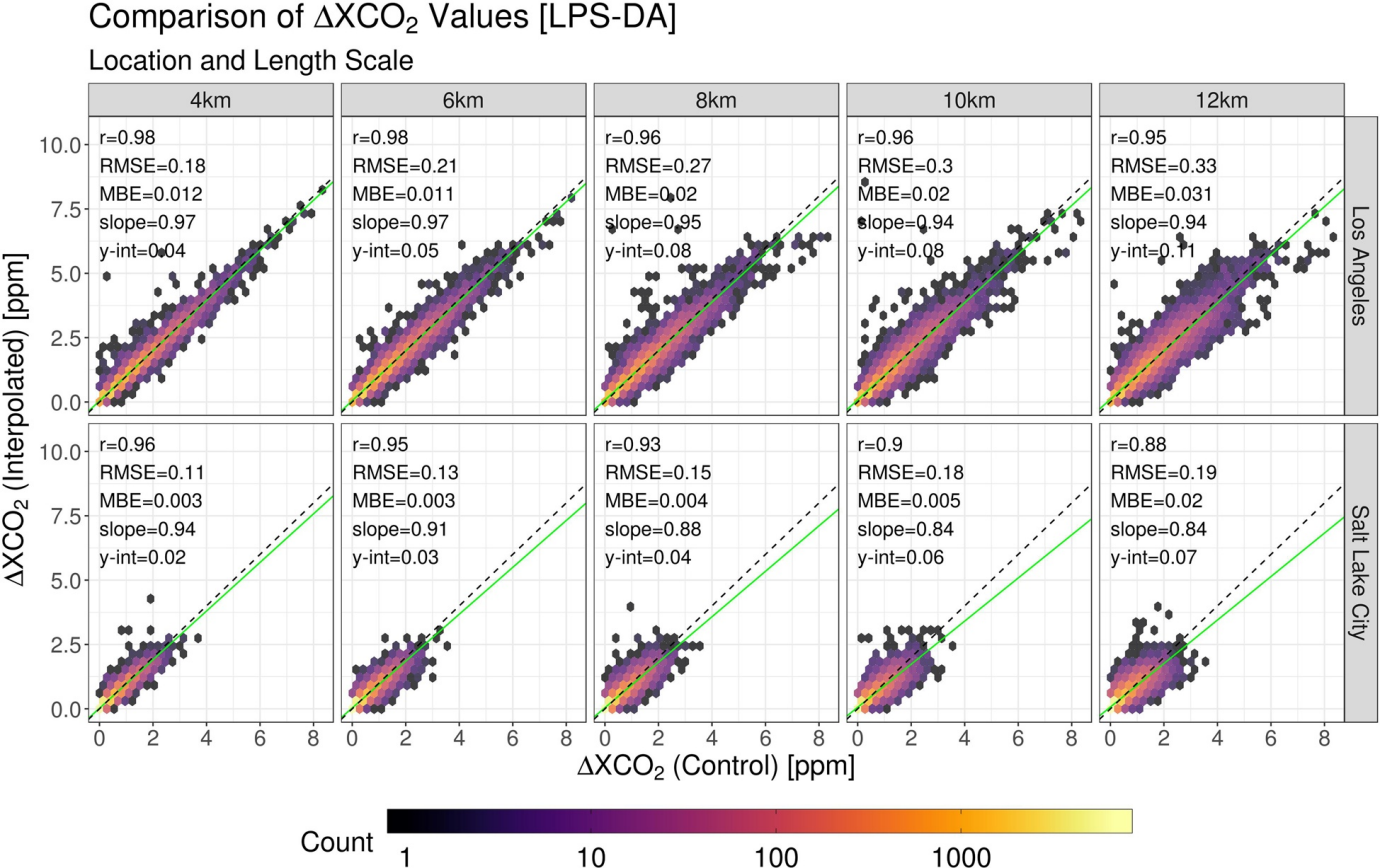
Stratified by location and subset length scale, s‐SAM ΔXCO_2_ values are compared to their interpolated counterparts after implementing a large point source detection algorithm. Each panel contains column‐averaged receptors from s‐SAMs representative of 15 days. Three times were sampled per day to incorporate diurnal features. These values are compared independently of their geographic location. The density of points within the comparisons are represented by the color gradient. The 1:1 ratio line is indicated by the black dashed line and the line of best fit is represented by the green line. Statistics from each fit are supplied in the top left corner of each panel. (SAM, snapshot area mapping).

Error statistics after the LPS‐DA was applied are presented in Figure 13. As with the previous data, there was a difference in the ranges of ΔXCO_2_ values between the two testing locations; however, the detection algorithm reduced the ranges at both sites. At the LA location, the largest ΔXCO_2_ values were removed with the remaining values only approaching 8 ppm. The SLC location experienced no profound reduction but ΔXCO_2_ values were constrained under 4ppm. Furthermore, correlation coefficients increased after the detection algorithm was applied. LA was most benefited as the correlation coefficients across length scales were increased by 0.02–0.09. The SLC location was less affected, with increases in correlation ranging from 0.00 – 0.02.

After applying the detection algorithm, RMSE values at LA were reduced by roughly half, reinforcing the notion that overall error is heavily influenced by LPS‐driven ΔXCO_2_ values. Conversely, applying the algorithm to the already small ΔXCO_2_ values and low number of LPSs present at the SLC had less of an impact on RMSE. The detection algorithm also reduced MBE in the two smallest length scales at the LA location. For larger length scales, the algorithm’s influence on MBE was ambiguous. The SLC location experienced a slight increase in MBE across all length scales. This is due to the removal of large, underestimated ΔXCO_2_ values during the detection process, increasing the influence of the over‐estimated yet smaller error values. Increasing slope values suggested that many of the receptors identified by the algorithm were large ΔXCO_2_ values that were underestimated by the interpolation method. Removing these points from the interpolation scheme applied more weight to the overestimated error associated with smaller ΔXCO_2_ values.

Results after applying the detection algorithm are presented in Table 5. Notable reductions in range were demonstrated as minimum and maximum values across all length scales were drastically reduced. Since most error values fall within the OCO‐2 error range and are centered near 0 ppm, their high‐density dominated the mean. Although most errors were relatively confined, the spread of these values was heavily influenced by outliers. After removal from the interpolation scheme, the SDs associated with the LA errors were reduced by roughly half and SD values at SLC were reduced by ∼0.02 ppm. The reduction in outliers is highlighted in the percentages of values lying outside the OCO‐2 and ±1 ppm ranges (“O.R. OCO‐2” and “O.R. 1ppm” in Table 5). LA experienced considerable reductions in the amount of outliers with O.R. OCO‐2 <5% for 4  and 6 km length scales. Reductions in O.R. 1 ppm such that O.R. 1 ppm < 1% for 4 , 6, and 8 km length scales were also noted. The larger range of OCO‐2 error associated with SLC allowed for the amount of all outlying values to be less than 2% across all length scales. Furthermore, O.R. 1 ppm was less than 1% for all SLC length scales.

**Table 5:** *Presented Here is a Summary of errors After a Large Point Source Detection Algorithm has Been Applied*

Location	Stat (in ppm)	OCO‐2 error	4 km	6 km	8 km	10 km	12 km
Los Angeles	Min	−0.48	−1.23	−1.77	−1.95	−2.10	−2.44
	Mean	0.00	0.01	0.01	0.02	0.02	0.03
	Max	0.48	4.70	2.18	6.35	8.23	4.56
	SD	0.28	0.18	0.21	0.27	0.30	0.33
	O.R. OCO‐2		2.36%	4.03%	6.98%	8.22%	11.36%
	O.R. 1ppm		0.29%	0.41%	0.97%	1.25%	2.00%
Salt Lake City	Min	−0.72	−0.97	−1.86	−1.54	−1.59	−1.94
	Mean	0.1	0.00	0.00	0.00	0.00	0.02
	Max	0.72	2.55	1.51	3.19	2.75	2.35
	SD	0.41	0.11	0.13	0.15	0.18	0.19
	O.R. OCO‐2		0.22%	0.40%	0.70%	1.26%	1.37%
	O.R. 1ppm		0.04%	0.10%	0.18%	0.38%	0.39%

The minimum (Min), maximum (Max), mean, and standard deviation (S.D.) are listed in ppm. The percentage of values lying outside of each location’s corresponding OCO‐2 error (O.R. OCO‐2) and the ±1 ppm range (O.R. 1 ppm) are also listed for each subset length scale. (O.R., out of range.)

W18 addresses errors from a variety of other sources. In their Riyadh test case, the per sounding error in modeled ΔXCO_2_ from atmospheric transport was reported to be 0.07–2.87 ppm for areas of low urban enhancement with an occasional value >5 ppm in areas of localized high urban enhancement. Using several CO_2_ emission inventories, W18 calculated a per sounding error range of 0.04–2.82 ppm for prior emission estimates (ODIAC). Additionally, the selection of parameters in X‐STILT was associated with ∼4% error in modeled ΔXCO_2_. Applying the LPS‐DA, the errors associated with the interpolation method in this work fell closely in line W18’s estimates of external errors. Across both locations, ≤2% of all error values had the potential of being out of the range associated with transport and prior emissions error. Given the analyses presented here, the most constraining source of error is the per sounding OCO‐2 errors.

### 3.6 Dependency on the Grid Spacing of Driving Meteorology

**Figure 14:**
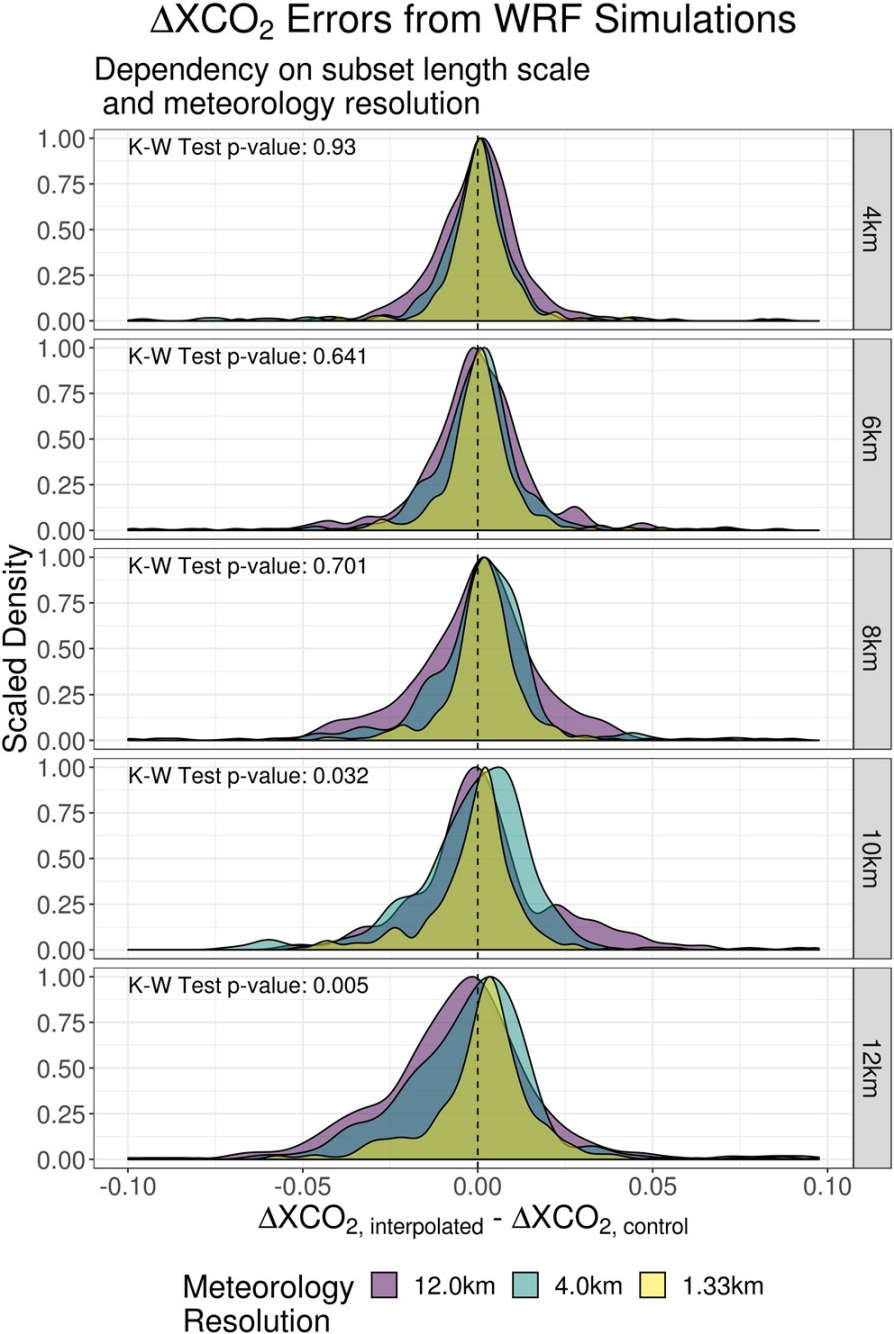
The distribution of errors presented here were generated using three different resolutions of WRF output for the Salt Lake City region: 1.33, 4.0, and 12.0 km. With each WRF domain, the interpolation method was applied using several subset length scales (4–12 km). The results were then stratified by WRF resolution and length scale. (WRF, Weather Research and Forecasting).

Using s‐SAMs generated from available WRF data for SLC (September and October of 2015), the influence of meteorology resolution was investigated. A Kruskal‐Wallis H test (see Section A2) was applied and the results are presented in Figure 14. The errors associated with the 1.33 km WRF resolution demonstrated the smallest increase in spread as the subset length scale increased; furthermore, these values maintained the smallest spread when compared to other resolutions at each length scale. With a resolution this fine, meteorological variables were interpolated across multiple points within all subset length scales. This allowed for potentially smoother changes in variable values across length scales and better agreement among control footprints used in the interpolation process. Conversely, when the most‐coarse meteorology field (12.0 km) was used, no subset extent was capable of containing more than one point of WRF interpolation. This coarse gridding likely induced abrupt changes in topography and meteorology.

Another feature of the data presented in Figure 14 was the tendency for the interpolation scheme to overestimate ΔXCO_2_ when a higher resolution WRF and larger subset length scale were used while interpolated values relying on the low resolution WRF data were underestimated. This reinforced topography’s influence on the interpolation results. Using the HRRR data, the use of large length scales at the SLC location resulted in a tendency of overestimation along mountain ranges (Figure 8). 1.33  and 4.0 km WRF data are also capable of resolving many of the features associated with these mountain ranges. The 12.0 km WRF data is less likely to capture these features, reducing their effects on interpolation results. Overall, when considering only 4–8 km length scales there were no statistically significant differences between the means of interpolation errors. Only at the larger length scales were differences significant. These differences were driven predominantly by the coarsening of the meteorological grid and the smoothing of topological features. *p*‐Values from the Kruskal‐Wallis test are included in Figure 14.

### 3.7 Interpolation Efficiency

**Figure 15:**
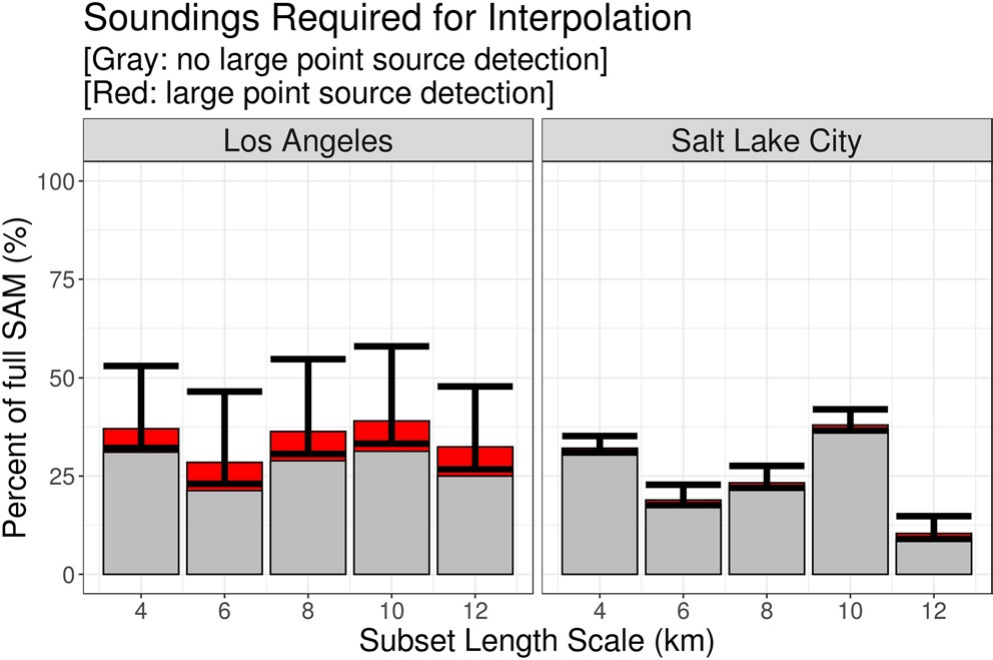
Implementing the interpolation method required the same number of control and interpolated receptors for all s‐SAMs. The percentages of required receptors (and control footprints) for each subset length scale is represented by the gray bars. After applying a large point source detection algorithm, the number of control and interpolated receptors varied due to day‐to‐day meteorological influences. The average number of required receptors/footprints after applying the algorithm is represented by the red bars. The minimum and maximum values of this range is represented by the black bars. The geometry of subsets and their interactions with the full s‐SAM domain may result in some receptor locations being excluded from subsets (notable examples are the 8  and 10 km length scales). These receptors as passed to the X‐STILT model. (SAM, snapshot area mapping; X‐STILT, Stochastic Time‐Inverted Lagrangian Transport).

Figure 15 presents the efficiency of the interpolation method throughout the two case studies. When the LPS‐DA was not applied, the 4 km length scale interpolation scheme consistently required ∼30% of the X‐STILT generated footprints to construct a full s‐SAM via interpolation. By increasing the length scale to 6 km, ∼25% of the receptors required an X‐STILT generated footprint to interpolate across the remainder of the s‐SAM. Without the LPS‐DA, these values remained unchanged across all s‐SAMs, as the number of receptors passed to X‐STILT did not change. These values are represented by the gray bars in the figure. Typically, as the subset length scale increases, the percentage of required X‐STILT generated footprints decreases. This is shown in the 4  and 6 km length scales but a different pattern exists in the 8  and 10 km length scales. Referring back to Figure 5, the subsets are defined from the bottom left corner; thus, the rightmost column and topmost row cannot fall within a complete subset. These receptors are passed to the X‐STILT model and footprints are generated using the traditional method. Similarly, all s‐SAMs in this work are broken into equal subsets beginning from the bottom left element of a full domain.

Due to certain geometries, it is possible for large sections on the edge of an s‐SAM to not be assigned a subset. The 8  and 10 km subset length scales highlight the importance of the s‐SAM domain and subset interaction and its consideration when implementing this process; however, in an idealized case where all soundings within a SAM domain are part of a subset, the 4, 6, 8, 10, and 12 km require 25.00%, 11.11%, 6.25%, 4.00%, and 2.78% of the footprints to be generated by X‐STILT. The remainder can be interpolated.

When the LPS‐DA was applied to each s‐SAM, the number of soundings removed from the interpolation process varied depending on meteorological factors. The footprints for soundings selected by the algorithm were generated by X‐STILT rather than interpolation. Applying the LPS‐DA yielded results that were dependent upon meteorological conditions. Across a duration of low atmospheric transport, emissions from LPSs are weakly diluted, increasing the likelihood of being detected by the LPS‐DA. In this case, more soundings will be identified by the algorithm and passed to X‐STILT, reducing the number of footprints to be interpolated. Conversely, over periods of strong transport, a smaller number of signals from LPSs may be detected. This will increase the number of footprints that can be generated via interpolation. These two scenarios are represented in Figure 15 by the black bars. The least efficient scenario (weak transport) is marked by the high end of the black bar. This scenario required the most X‐STILT generated footprints, reducing the speed gained from the interpolation scheme. The low end of the black bar represents the most efficient scenario (strong mixing/transport) in which the LPS‐DA was applied. The average efficiency across all s‐SAMs is represented by the red bar.

Computations in this work were distributed across a bank of 8‐core Intel Xeon E5‐2670 2.6 GHz processors; however, small time trials were performed on sequential calculations to assess the difference in run time between X‐STILT generated and interpolated footprints. A single X‐STILT generated footprint using 100 particles per vertical level averaged a run time of 11 min and 37 s (*n* = 7). The generation of interpolated footprints averaged a run time of 23 s (*n* = 12). Thus, each interpolated footprint was generated in ∼3% of the time X‐STILT required. There was considerable variance among run times for the interpolation process due to the number of footprints required in each interpolation. Interpolated footprints along the periphery of subsets required only two control footprints whereas interior locations required four. This doubled the number of raster files to be manipulated in the interpolation process, leading to longer run times.

For an estimation of the computational time required to interpolate a full SAM, an approximation was made with the following assumptions: (1) five nodes consisting of 10 cores (E5‐2670 2.6 GHz processors) were available, (2) X‐STILT footprints are created first, followed by interpolations, and (3) both X‐STILT and the interpolation scheme were capable of running in parallel. Given the distribution of errors and density of LPSs at each location, a 4 km subset length scale was selected for LA. A 6 km length scale was selected for SLC. After applying the LPS‐DA across all s‐SAMs, the average number of receptors passed to X‐STILT at the LA location was 171 (of 460) while SLC only required an average of 95 (of 500). Assuming computations were distributed evenly, the estimated time required to generate footprints for an entire s‐SAM was reduced by 62% and 78%, respectively.

## 4 Discussion

The number of space‐based CO_2_ observation platforms is projected to increase and subsequent increases in instrument resolution is expected to follow. With the anticipation of spatially dense XCO_2_ measurements on the horizon, the development of an interpolation scheme is needed, aimed to reduce the burden on the X‐STILT model as these data are digested and analyzed. Estimates suggested that, on average, reductions of 62% and 78% in computational time can be attained at the Los Angeles and Salt Lake City test locations. Errors accrued by this method remained largely within the range of external errors (instrument retrieval error, prior emissions error, atmospheric transport error, etc.) at both test sites. Regional meteorology and topography influenced the accuracy of the method but most effects could be mitigated by careful selection of a subset length scale. These effects and the effects of LPSs were mitigated by selecting a smaller length scale that also constrained interpolation errors within the associated instrument error. A 6 km subset length scale was selected for the SLC location and a 4 km subset length scale was selected for LA.

Currently, this interpolation method must be optimized on a site‐by‐site basis. Recommendations for the two case studies in this work may not be broadly applicable. For new locations of interest, the generation of several preliminary s‐SAMs will be required. The generated s‐SAMs should cover several times of day and multiple seasons. Modeled values can be systematically removed to test several subset length scales. Based on (1) the distributions of LPS locations, (2) sensitivity to topographic features, (3) interpolated ΔXCO_2_ errors, and (4) the error of the instrument(s) used for XCO_2_ observation, an appropriate subset length scale can be selected. Future users may generate an initial coarse sampling of s‐SAMs to calculate performance statistics, adding additional s‐SAMs as needed. The required sensitivity analyses and subsequent “tuning” of the interpolation scheme for each location of interest makes broad implementation difficult and limits the use of this method to areas of intensive and long‐term study.

Moving forward, a dynamic detection algorithm can be implemented. In this work, the detection algorithm aimed to detect large point sources and remove nearby receptors from the interpolation scheme. Although results show reasonable effectiveness, influences from meteorology or topography are not considered. More rigorously designed algorithms may incorporate methods to detect areas of potentially large error based on these factors. Schemes where the subset length scale is varied across SAMs may also be implemented. Expanding X‐STILT to a regional model would allow for the dissection of regional XCO_2_ domains by investigating the individual footprints corresponding to key ΔXCO_2_ values. Although a coarse analysis was presented in this work, future investigations can integrate this interpolation scheme with emission inventories of high temporal resolution (Nassar et al., 2013) and biospheric CO_2_ source/sink inventories. Additional inputs will provide computationally efficient and accurate simulated XCO_2_ values for comparison with OCO‐3 SAMs or future high‐density XCO_2_ retrieval missions such as NASA’s Geostationary Carbon Observatory (GeoCarb) (Moore et al., 2018) or the ESA’s Copernicus Anthropogenic Carbon Dioxide Monitoring instrument (CO2M) (Kuhlmann et al., 2019).

## 5 Conclusions

Space‐based XCO_2_ observations are projected to increase in the coming years. One of the most recent sensors, NASA’s OCO‐3 instrument, is set to provide unprecedented spatial and temporal coverage of targeted areas around the Earth. Consequently, a strain on current analysis methods is expected. In preparation for unparalleled data availability, this work presents a novel interpolation method for influence footprints derived from the column‐averaged (“X”) Stochastic Time‐Inverted Lagrangian Transport (X‐STILT) model. The traditional X‐STILT approach requires rigorous computation to determine the upwind influences associated with each OCO‐3 sounding. Here, a series of simulated OCO‐3 observations were generated to evaluate the effectiveness of the interpolation scheme. The methodology used a key subset of OCO‐3 soundings and their associated influence footprints generated by X‐STILT. Using a nonlinear weighted averaging, synthetic footprints were created for the remaining sounding locations. The relationship between decreasing the number of X‐STILT generated footprints and interpolation accuracy was thoroughly investigated along with the influences of meteorology, topography, and meteorology resolution. The influences of large point sources were given special attention via a filtering scheme. In two test cases: Los Angeles, CA and Salt Lake City, UT, time trials revealed an estimated 62% and 78% reduction in computational time when compared to the traditional X‐STILT modeling approach. Errors associated with the interpolation method remained within the range of external errors (instrument error, atmospheric transport error, prior emissions estimates, etc.), demonstrating the viability of this method to reduce the computational time required of dense observations of space‐based XCO_2_ and other space‐based trace gas measurements.

## Data Availability

Relevant OCO‐2 data were publicly provided by NASA’s OCO‐2 project and retrieved from the Goddard Earth Science Data and information Services Center (https://disc.gsfc.nasa.gov/datasets/OCO2_L2_Lite_FP_9r/summary, DOI: 10.5067/W8QGIYNKS3JC). The HRRR data used in this work were downloaded from the National Oceanic and Atmospheric Administration’s (NOAA) Air Resources Laboratory (ARL) and is also publicly available at https://www.ready.noaa.gov/archives.php. Additionally, current and previous versions of the ODIAC dataset are maintained by the Center for Global Environmental Research and can be accessed via: http://db.cger.nies.go.jp/dataset/ODIAC/. Details are provided in Oda et al. (2018). Source code for X‐STILT can be found at https://doi.org/10.5281/zenodo.2556989. Lastly, the authors of this work declare no conflicts of interest.

## Appendix A Metrics

### A1 Threat Score (TS) & Weighted Mean Absolute Error (WMAE)

The process of calculating the threat score for two footprints at a column‐based receptor location requires the construction of two binary arrays: *F*_*C*,bin_(*x*_*i*_, *y*_*j*_) and *F*_*I*,bin_(*x*_*i*_, *y*_*j*_). Letting *f*_*C*_ (*x*_*i*_, *y*_*j*_, *t*_0_) ∈ *F*_*C*_ (*λ*, *ϕ*, *t*_0_) and $\hat{f}\left(x_{i},y_{j},t_{0}\right)\in \hat{F}\left(\lambda ,\phi ,t_{0}\right)$ represent the elements of a control footprint and the elements of an interpolated footprint, respectively, the two arrays are constructed such that:

(A1) $$F_{C,bin}\left(x_{i},y_{j}\right)=\{\begin{array}{cc} 1\; & f_{C}\left(x_{i},y_{j},t_{0}\right)>0\\ 0\; & f_{C}\left(x_{i},y_{j},t_{0}\right)=0 \end{array}$$

and

(A2) $$F_{I,bin}\left(x_{i},y_{j}\right)=\{\begin{array}{cc} 1\; & \hat{f}\left(x_{i},y_{j},t_{0}\right)>0\\ 0\; & \hat{f}\left(x_{i},y_{j},t_{0}\right)=0 \end{array}$$

These binary arrays are first used to calculate a crucial component of the threat score: the spatial agreement between both footprints. The number of elements contained in the intersection, |*F*_*C*,*bin*_(*x*_*i*_, *y*_*i*_) ∩ *F*_*I*,*bin*_(*x*_*i*_, *y*_*i*_)|, is determined by the total number of elements where *F*_*C*,*bin*_(*x*_*i*_, *y*_*i*_) + *F*_*I*,*bin*_(*x*_*i*_, *y*_*i*_) = 2. This value is then used in tandem with the total number of nonzero values in each separate array to determine the threat score (TS):

(A3) $$TS=\frac{\left|F_{C,bin}(x_{i},y_{i})\cap F_{I,bin}(x_{i},y_{i})\right|}{\sum_{ij}F_{C,bin}(x_{i},y_{i})+\sum_{ij}F_{I,bin}(x_{i},y_{i})-|F_{C,bin}(x_{i},y_{i})\cap F_{I,bin}(x_{i},y_{i})|}\times 100\%.$$

This score provides a percentage of spatial agreement between the two footprints; furthermore, Equation A1 is used in the calculation of WMAE such that

(A4) $$WMAE=\left(\frac{1}{\sum_{ij}F_{C,bin}(x_{i},y_{i})}\right)\left(\sum_{ij}F_{C,bin}(x_{i},y_{i})\cdot |\hat{f}(x_{i},y_{j},t_{0})-f_{C}(x_{i},y_{j},t_{0})|\right).$$

Including the weighting term removes any error calculations where there is spatial mismatch between the control and interpolated footprint, allowing for strict evaluation of the interpolation method’s ability to produce similar element values at control points.

### A2. Kruskal‐Wallis H Test

This test serves as a nonparametric alternative to the Analysis of Variance (ANOVA) test using a rank‐based approach (Kruskal & Wallis, 1952). The initial step in using this test required errors within each group (WRF resolutions) to be ordered in ascending order. An *H* value was then calculated by

(A5) $$H=\frac{12}{N\left(N+1\right)}\overset{C}{\underset{i=1}{\sum }}\frac{R_{i}^{2}}{n_{i}}-3\left(N+1\right).$$

*C* is the total number of groups (*C* = 3), *n*_*i*_ is the number of elements in group, and *R*_*i*_ is the average rank of the values in each group. In a Kruskal‐Wallis H test, the rank of each value is determined by listing all samples, *N*, in ascending order and assigning a numerical rank (1, 2, …, *N*) to each value. *H* approximately follows a *χ* ^2^ distribution, allowing for the calculation of a *p*‐value.

## References

[ess2795-bib-0001] Apte, J. S. , Messier, K. P. , Gani, S. , Brauer, M. , Kirchstetter, T. W. , Lunden, M. M. , & Hamburg, S. P. (2017). High‐resolution air pollution mapping with Google street view cars: Exploiting big data. Environmental Science & Technology, 51(12), 6999–7008. 10.1021/acs.est.7b00891 28578585

[ess2795-bib-0002] Bares, R. , Mitchell, L. , Fasoli, B. , Bowling, D. , Catharine, D. , Garcia, M. , & Lin, J. C. (2019). The Utah urban carbon dioxide (UUCON) and Uintah Basin greenhouse gas networks: Instrumentation, data and measurement uncertainty. Earth System Science Data Discussions, 11, 1291–1308. 10.5194/essd-11-1291-2019

[ess2795-bib-0003] Benjamin, S. G. , Weygandt, S. S. , Brown, J. M. , Hu, M. , Alexander, C. R. , Smirnova, T. G. , & Manikin, G. S. (2016). A north American hourly assimilation and model forecast cycle: The rapid refresh. Monthly Weather Review, 144(4), 1669–1694. 10.1175/mwr-d-15-0242.1

[ess2795-bib-0004] Bush, S. E. , Hopkins, F. M. , Randerson, J. T. , Lai, C.‐T. , & Ehleringer, J. R. (2015). Design and application of a mobile ground‐based observatory for continuous measurements of atmospheric trace gas and criteria pollutant species. Atmospheric Measurement Techniques, 8(8), 3481–3492. 10.5194/amt-8-3481-2015

[ess2795-bib-0005] Connor, B. , Bösch, H. , McDuffie, J. , Taylor, T. , Fu, D. , Frankenberg, C. , & Jiang, Y. (2016). Quantification of uncertainties in OCO‐2 measurements of XCO2: Simulations and linear error analysis. Atmospheric Measurement Techniques, 9(10), 5227–5238. 10.5194/amt-9-5227-2016

[ess2795-bib-0006] Davidson, K. , Coenen, L. , & Gleeson, B. (2019). A decade of c40: Research insights and agendas for city networks. Global Policy, 10(4), 697–708. 10.1111/1758-5899.12740

[ess2795-bib-0007] Dietrich, F. , Chen, J. , Voggenreiter, B. , Aigner, P. , Nachtigall, N. , & Reger, B. (2021). MUCCnet: Munich urban carbon column network. Atmospheric Measurement Techniques, 14(2), 1111–1126. 10.5194/amt-14-1111-2021

[ess2795-bib-0008] Eldering, A. , Boland, S. , Solish, B. , Crisp, D. , Kahn, P. , & Gunson, M. (2012). High precision atmospheric Co_2_ measurements from space: The design and implementation of OCO‐2. In 2012 IEEE aerospace conference. New York, NY: Institute of Electrical and Electronics Engineers. 10.1109/aero.2012.6187176

[ess2795-bib-0009] Eldering, A. , Taylor, T. E. , O’Dell, C. W. , & Pavlick, R. (2019). The OCO‐3 mission: Measurement objectives and expected performance based on 1 year of simulated data. Atmospheric Measurement Techniques, 12(4), 2341–2370. 10.5194/amt-12-2341-2019

[ess2795-bib-0010] Fasoli, B. , Lin, J. C. , Bowling, D. R. , Mitchell, L. , & Mendoza, D. (2018). Simulating atmospheric tracer concentrations for spatially distributed receptors: updates to the Stochastic Time‐Inverted Lagrangian Transport model’s R interface (STILT‐R version 2). Geoscientific Model Development, 11(7), 2813–2824. 10.5194/gmd-11-2813-2018

[ess2795-bib-0011] Hedelius, J. K. , Liu, J. , Oda, T. , Maksyutov, S. , Roehl, C. M. , Iraci, L. T. , & Wennberg, P. O. (2018). Southern California megacity CO_2_, CH_4_, and CO flux estimates using ground‐ and space‐based remote sensing and a Lagrangian model. Atmospheric Chemistry and Physics, 18(22), 16271–16291. 10.5194/acp-18-16271-2018

[ess2795-bib-0012] Hogue, S. , Marland, E. , Andres, R. J. , Marland, G. , & Woodard, D. (2016). Uncertainty in gridded CO_2_ emissions estimates. Earth’s Future, 4(5), 225–239. 10.1002/2015ef000343

[ess2795-bib-0013] Hogue, S. , Roten, D. , Marland, E. , Marland, G. , & Boden, T. A. (2017). Gridded estimates of CO_2_ emissions: Uncertainty as a function of grid size. Mitigation and Adaptation Strategies for Global Change, 24(6), 969–983. 10.1007/s11027-017-9770-z

[ess2795-bib-0014] Hutchins, M. G. , Colby, J. D. , Marland, G. , & Marland, E. (2016). A comparison of five high‐resolution spatially‐explicit, fossil‐fuel, carbon dioxide emission inventories for the united states. Mitigation and Adaptation Strategies for Global Change, 22(6), 947–972. 10.1007/s11027-016-9709-9

[ess2795-bib-0015] Jacobson, M. Z. (April 2010). Enhancement of local air pollution by urban CO_2_ domes. Environmental Science & Technology, 44(7), 2497–2502. 10.1021/es903018m 20218542

[ess2795-bib-0016] Janardanan, R. , Maksyutov, S. , Oda, T. , Saito, M. , Kaiser, J. W. , Ganshin, A. , & Yokota, T. (2016). Comparing GOSAT observations of localized CO_2_ enhancements by large emitters with inventory‐based estimates. Geophysical Research Letters, 43, 3486–3493. 10.1002/2016gl067843

[ess2795-bib-0017] Jolliffe , I. T. , & Stephenson D. B. (Eds.) (2003). Forecast verification: A practitioner’s guide in atmospheric science. New York: John Wiley.

[ess2795-bib-0018] Kahle, D. , & Wickham, H. (2013). ggmap: Spatial visualization with ggplot2. The R Journal, 5(1), 144–161. https://journal.r-project.org/archive/2013-1/kahle-wickham.pdf

[ess2795-bib-0019] Keeling, R. F. , & Keeling, C. D. (2017). Atmospheric monthly in situ CO_2_ data—Mauna loa observatory, Hawaii. In Scripps CO_2_ program data. La Jolla, CA: UC San Diego Library Digital Collections. 10.6075/J08W3BHW

[ess2795-bib-0020] Kruskal, W. H. , & Wallis, W. A. (1952). Use of ranks in one‐criterion variance analysis. Journal of the American Statistical Association, 47(260), 583–621. 10.1080/01621459.1952.10483441

[ess2795-bib-0021] Kuhlmann, G. , Broquet, G. , Marshall, J. , Clément, V. , Löscher, A. , Meijer, Y. , & Brunner, D. (2019). Detectability of CO_2_ emission plumes of cities and power plants with the copernicus anthropogenic CO_2_ monitoring (CO_2_m) mission. Atmospheric Measurement Techniques, 12(12), 6695–6719. 10.5194/amt-12-6695-2019

[ess2795-bib-0022] Kunik, L. , Mallia, D. V. , Gurney, K. R. , Mendoza, D. L. , Oda, T. , & Lin, J. C. (2019). Bayesian inverse estimation of urban CO_2_ emissions: Results from a synthetic data simulation over Salt Lake City, UT. Elementa: Science of the Anthropocene, 7(1), 36. 10.1525/elementa.375

[ess2795-bib-0023] Labzovskii, L. D. , Jeong, S.‐J. , & Parazoo, N. C. (2019). Working toward confident spaceborne monitoring of carbon emissions from cities using orbiting carbon observatory‐2. Remote Sensing of Environment, 233, 111359. 10.1016/j.rse.2019.111359

[ess2795-bib-0024] Le Quéré, C. , Raupach, M. R. , Canadell, J. G. , Marland, G. , Bopp, L. , Ciais, P. , & Woodward, F. I. (2009). Trends in the sources and sinks of carbon dioxide. Nature Geoscience, 2(12), 831–836. 10.1038/ngeo689

[ess2795-bib-0025] Lin, J. C. , Gerbig, C. , Wofsy, S. C. , Andrews, A. E. , Daube, B. C. , Davis, K. J. , & Grainger, C. A. (2003). A near‐field tool for simulating the upstream influence of atmospheric observations: The stochastic time‐inverted Lagrangian transport (stilt) model. Journal of Geophysical Research: Atmospheres, 108(D16), 4493. 10.1029/2002JD003161

[ess2795-bib-0026] Lin, J. C. , Mallia, D. V. , Wu, D. , & Stephens, B. B. (2017). How can mountaintop CO_2_ observations be used to constrain regional carbon fluxes?. Atmospheric Chemistry and Physics, 17(9), 5561–5581. 10.5194/acp-17-5561-2017

[ess2795-bib-0027] Lin, J. C. , Mitchell, L. , Crosman, E. , Mendoza, D. L. , Buchert, M. , Bares, R. , & Ehleringer, J. (2018). CO_2_ and carbon emissions from cities: Linkages to air quality, socioeconomic activity, and stakeholders in the Salt Lake City urban area. Bulletin of the American Meteorological Society, 99(11), 2325–2339. 10.1175/bams-d-17-0037.1

[ess2795-bib-0028] Liu, Y. , Wang, J. , Yao, L. , Chen, X. , Cai, Z. , Yang, D. , & Lyu, D. (2018). The Tansat mission: Preliminary global observations. Science Bulletin, 63(18), 1200–1207. 10.1016/j.scib.2018.08.004 36751089

[ess2795-bib-0029] Mallia, D. V. , Lin, J. C. , Urbanski, S. , Ehleringer, J. , & Nehrkorn, T. (2015). Impacts of upwind wildfire emissions on CO, CO_2_, and PM2.5 concentrations in Salt Lake City, Utah. Journal of Geophysical Research: Atmospheres, 120(1), 147–166. 10.1002/2014jd022472

[ess2795-bib-0030] Matthews, H. D. , Graham, T. L. , Keverian, S. , Lamontagne, C. , Seto, D. , & Smith, T. J. (2014). National contributions to observed global warming. Environmental Research Letters, 9(1), 014010. 10.1088/1748-9326/9/1/014010

[ess2795-bib-0031] Mitchell, L. E. , Crosman, E. T. , Jacques, A. A. , Fasoli, B. , Leclair‐Marzolf, L. , Horel, J. , & Lin, J. C. (2018). Monitoring of greenhouse gases and pollutants across an urban area using a light‐rail public transit platform. Atmospheric Environment, 187, 9–23. 10.1016/j.atmosenv.2018.05.044

[ess2795-bib-0032] Mitchell, L. E. , Lin, J. C. , Bowling, D. R. , Pataki, D. E. , Strong, C. , Schauer, A. J. , & Ehleringer, J. R. (2018). Long‐term urban carbon dioxide observations reveal spatial and temporal dynamics related to urban characteristics and growth. Proceedings of the national Academy of Sciences of the United Stated of America, 115(12), 2912–2917. 10.1073/pnas.1702393115 PMC5866532 29507190

[ess2795-bib-0033] Moore, B. , Crowell, S. M. R. , Rayner, P. J. , Kumer, J. , O’Dell, C. W. , O’Brien, D. , & Lemen, J. (2018). The potential of the geostationary carbon cycle observatory (GeoCarb) to provide multi‐scale constraints on the carbon cycle in the Americas. Frontiers in Environmental Science, 6, 109. 10.3389/fenvs.2018.00109

[ess2795-bib-0034] Nassar, R. , Hill, T. G. , McLinden, C. A. , Wunch, D. , Jones, D. B. A. , & Crisp, D. (2017). Quantifying CO_2_ emissions from individual power plants from space. Geophysical Research Letters, 44, 10045–10053. 10.1002/2017gl074702

[ess2795-bib-0035] Nassar, R. , Napier‐Linton, L. , Gurney, K. R. , Andres, R. J. , Oda, T. , Vogel, F. R. , & Deng, F. (2013). Improving the temporal and spatial distribution of CO_2_ emissions from global fossil fuel emission data sets. Journal of Geophysical Research: Atmospheres, 118(2), 917–933. 10.1029/2012JD018196

[ess2795-bib-0036] Nehrkorn, T. , Eluszkiewicz, J. , Wofsy, S. C. , Lin, J. C. , Gerbig, C. , Longo, M. , & Freitas, S. (2010). Coupled weather research and forecasting–stochastic time‐inverted Lagrangian transport (WRF–STILT) model. Meteorology and Atmospheric Physics, 107(1–2), 51–64. 10.1007/s00703-010-0068-x

[ess2795-bib-0037] Oda, T. , Bun, R. , Kinakh, V. , Topylko, P. , Halushchak, M. , Marland, G. , & Horabik‐Pyzel, J. (2019). Errors and uncertainties in a gridded carbon dioxide emissions inventory. Mitigation and Adaptation Strategies for Global Change, 24(6), 1007–1050. 10.1007/s11027-019-09877-2

[ess2795-bib-0038] Oda, T. , & Maksyutov, S. (2011). A very high‐resolution (1km × 1km) global fossil fuel CO_2_ emission inventory derived using a point source database and satellite observations of nighttime lights. Atmospheric Chemistry and Physics, 11(2), 543–556. 10.5194/acp-11-543-2011

[ess2795-bib-0039] Oda, T. , Maksyutov, S. , & Andres, R. J. (2018). The open‐source data inventory for anthropogenic CO_2_, version 2016 (ODIAC 2016): a global monthly fossil fuel CO_2_ gridded emissions data product for tracer transport simulations and surface flux inversions. Earth System Science Data, 10(1), 87–107. 10.5194/essd-10-87-2018 31662803 PMC6818511

[ess2795-bib-0040] Richardson, S. J. , Miles, N. L. , Davis, K. J. , Lauvaux, T. , Martins, D. K. , Turnbull, J. C. , & Cambaliza, M. O. L. (October 2017). Tower measurement network of in‐situ CO_2_, CH_4_, and CO in support of the Indianapolis FLUX (INFLUX) Experiment. Elementa: Science of the Anthropocene, 5, 59. 10.1525/elementa.140

[ess2795-bib-0041] Rolph, G. , Stein, A. , & Stunder, B. (2017). Real‐time environmental applications and display sYstem: READY. Environmental Modelling & Software, 95, 210–228. 10.1016/j.envsoft.2017.06.025

[ess2795-bib-0042] Shusterman, A. A. , Teige, V. E. , Turner, A. J. , Newman, C. , Kim, J. , & Cohen, R. C. (2016). The Berkeley atmospheric co_2_ observation network: initial evaluation. Atmospheric Chemistry and Physics, 16(21), 13449–13463. 10.5194/acp-16-13449-2016

[ess2795-bib-0043] Singer, A. M. , Branham, M. , Hutchins, M. G. , Welker, J. , Woodard, D. L. , Badurek, C. A. , & Marland, G. (2014). The role of CO_2_ emissions from large point sources in emissions totals, responsibility, and policy. Environmental Science & Policy, 44, 190–200. 10.1016/j.envsci.2014.08.001

[ess2795-bib-0044] Tian, J. , Shan, Y. , Zheng, H. , Lin, X. , Liang, X. , & Guan, D. (2019). Structural patterns of city‐level CO_2_ emissions in northwest china. Journal of Cleaner Production, 223, 553–563. 10.1016/j.jclepro.2019.03.146

[ess2795-bib-0045] United Nations Human Settlements Programme . (2011). Cities and climate change: Global report on human settlements, 2011. London, UK: Earthscan.

[ess2795-bib-0046] Verhulst, K. R. , Karion, A. , Kim, J. , Salameh, P. K. , Keeling, R. F. , Newman, S. , & Miller, C. E. (2017). Carbon dioxide and methane measurements from the los angeles megacity carbon project. Part 1: Calibration, urban enhancements, and uncertainty estimates. Atmospheric Chemistry and Physics, 17(13), 8313–8341. 10.5194/acp-17-8313-2017 PMC6459414 30984251

[ess2795-bib-0047] Wang, G. , & Ostoja‐Starzewski, M. (2004). Influence of topography on the Phoenix CO_2_ dome: A computational study. Atmospheric Science Letters, 5(5), 103–107. http://doi.wiley.com/10.1002/asl.67

[ess2795-bib-0048] Wu, D. , Lin, J. C. , Fasoli, B. , Oda, T. , Ye, X. , Lauvaux, T. , & Kort, E. A. (2018). A Lagrangian approach toward extracting signals of urban co_2_ emissions from satellite observations of atmospheric column CO_2_ (XCO_2_): X‐stochastic time‐inverted Lagrangian transport model (x‐stilt v1). Geoscientific Model Development, 11(12), 4843–4871. 10.5194/gmd-11-4843-2018

[ess2795-bib-0049] Wu, D. , Lin, J. C. , Oda, T. , & Kort, E. A. (2020). Space‐based quantification of per capita CO_2_ emissions from cities. Environmental Research Letters, 15(3), 035004. 10.1088/1748-9326/ab68eb

[ess2795-bib-0050] Wunch, D. , Wennberg, P. O. , Osterman, G. , Fisher, B. , Naylor, B. , Roehl, C. M. , & Eldering, A. (2017). Comparisons of the orbiting carbon observatory‐2 (OCO‐2) CO_2_ measurements with TCCON. Atmospheric Measurement Techniques, 10(6), 2209–2238. 10.5194/amt-10-2209-2017

[ess2795-bib-0051] Xueref‐Remy, I. , Dieudonné, E. , Vuillemin, C. , Lopez, M. , Lac, C. , Schmidt, M. , & Ampe, C. (2018). Diurnal, synoptic and seasonal variability of atmospheric co_2_ in the paris megacity area. Atmospheric Chemistry and Physics, 18(5), 3335–3362. 10.5194/acp-18-3335-2018

[ess2795-bib-0052] Ye, X. , Lauvaux, T. , Kort, E. A. , Oda, T. , Feng, S. , Lin, J. C. , & Wu, D. (2020). Constraining fossil fuel CO_2_ emissions from urban area using OCO‐2 observations of total column CO_2_ . Journal of Geophysical Research: Atmospheres, 125, e2019JD030528. 10.1029/2019jd030528

[ess2795-bib-0053] Yokota, T. , Yoshida, Y. , Eguchi, N. , Ota, Y. , Tanaka, T. , Watanabe, H. , & Maksyutov, S. (2009). Global concentrations of CO_2_ and CH_4_ retrieved from GOSAT: First preliminary results. SOLA, 5, 160–163. 10.2151/sola.2009-041

[ess2795-bib-0054] Yue, T. , Zhang, L. , Zhao, M. , Wang, Y. , & Wilson, J. (2016). Space‐ and ground‐based CO_2_ measurements: A review. Science China Earth Sciences, 59(11), 2089–2097. 10.1007/s11430-015-0239-7

